# An 18 kDa Scaffold Protein Is Critical for *Staphylococcus epidermidis* Biofilm Formation

**DOI:** 10.1371/journal.ppat.1004735

**Published:** 2015-03-23

**Authors:** Rahel Decker, Christoph Burdelski, Melanie Zobiak, Henning Büttner, Gefion Franke, Martin Christner, Katharina Saß, Bernd Zobiak, Hanae A. Henke, Alexander R. Horswill, Markus Bischoff, Stephanie Bur, Torsten Hartmann, Carolyn R. Schaeffer, Paul D. Fey, Holger Rohde

**Affiliations:** 1 Institut für Medizinische Mikrobiologie, Virologie und Hygiene, Hamburg, Germany; 2 UKE Microscopy Imaging Facility, Universitätsklinikum Hamburg-Eppendorf, Hamburg, Germany; 3 Department of Microbiology, Carver College of Medicine, University of Iowa, Iowa City, Iowa, United States of America; 4 Institut für Medizinische Mikrobiologie und Hygiene, Universitätsklinikum des Saarlandes, Homburg, Germany; 5 Department of Pathology and Microbiology, Center for Staphylococcal Research, University of Nebraska Medical Center, Omaha, Nebraska, United States of America; National Institutes of Health, UNITED STATES

## Abstract

Virulence of the nosocomial pathogen *Staphylococcus epidermidis* is crucially linked to formation of adherent biofilms on artificial surfaces. Biofilm assembly is significantly fostered by production of a bacteria derived extracellular matrix. However, the matrix composition, spatial organization, and relevance of specific molecular interactions for integration of bacterial cells into the multilayered biofilm community are not fully understood. Here we report on the function of novel 18 kDa Small basic protein (Sbp) that was isolated from *S*. *epidermidis* biofilm matrix preparations by an affinity chromatographic approach. Sbp accumulates within the biofilm matrix, being preferentially deposited at the biofilm–substratum interface. Analysis of Sbp-negative *S*. *epidermidis* mutants demonstrated the importance of Sbp for sustained colonization of abiotic surfaces, but also epithelial cells. In addition, Sbp promotes assembly of *S*. *epidermidis* cell aggregates and establishment of multilayered biofilms by influencing polysaccharide intercellular-adhesin (PIA) and accumulation associated protein (Aap) mediated intercellular aggregation. While inactivation of Sbp indirectly resulted in reduced PIA-synthesis and biofilm formation, Sbp serves as an essential ligand during Aap domain-B mediated biofilm accumulation. Our data support the conclusion that Sbp serves as an *S*. *epidermidis* biofilm scaffold protein that significantly contributes to key steps of surface colonization. Sbp-negative *S*. *epidermidis* mutants showed no attenuated virulence in a mouse catheter infection model. Nevertheless, the high prevalence of *sbp* in commensal and invasive *S*. *epidermidis* populations suggests that Sbp plays a significant role as a co-factor during both multi-factorial commensal colonization and infection of artificial surfaces.

## Author Summary

Biofilm formation is a key phenotype allowing the otherwise harmless skin commensal *S*. *epidermidis* to establish chronic implant-associated infections, affecting millions of patients worldwide. *S*. *epidermidis* biofilm assembly relies on the production of an extracellular matrix that serves as glue to stabilize the multilayered bacterial architecture. Here we identified novel 18 kDa Small basic protein (Sbp) as a key component of the extracellular matrix that promotes pivotal steps of bacterial biofilm formation *in vitro*. Importantly, Sbp is deposited specifically at the interface between biofilm and substrate, as well as in larger humps interspersed within the bacterial cell architecture, thereby forming a proteinaceous biofilm scaffold. This localization enables Sbp to foster stable *S*. *epidermidis* interactions with an artificial surface and also contributes to *S*. *epidermidis* cell aggregation mechanisms, i.e., polysaccharide intercellular adhesin (PIA) and accumulation associated protein (Aap). In fact, by demonstrating direct Sbp-Aap interactions we provide the first evidence supporting the idea that specific molecular interactions between *S*. *epidermidis* and matrix components are involved in *S*. *epidermidis* biofilm accumulation. In conclusion, we here show that Sbp promotes key phenotypic features important for *S*. *epidermidis* to evolve as an opportunistic pathogen.

## Introduction

Hospital acquired infections are a major threat in modern medicine causing significant morbidity and mortality especially in high risk populations, e.g. old or immunosuppressed patients [1–4]. A major risk factor predisposing patients to develop infectious complications are medical procedures involving the permanent or intermittent implantation of artificial devices, i.e. central venous catheters, artificial heart valves, or joint prosthesis. The burden of over 400,000 cases per year in the US alone underscores the tremendous importance of implant associated infections [5,6], and it is estimated the 250,000–500,000 primary bloodstream infections related to intravascular devices cause attributable excess costs of up to $56,000 US dollars per episode [6]. Intriguingly, up to 80% of device associated infections are caused by otherwise harmless skin commensals belonging to the group of Coagulase-negative staphylococci, most notably *Staphylococcus epidermidis* [5,7]. Electron microscopy studies showed that on *ex vivo* catheters *S*. *epidermidis* organizes adherent, multi-cellular aggregates termed biofilms [8]. There is clear evidence from animal models of foreign-material infections that the ability to form a biofilm is of essential importance for pathogenicity of the species [9,10]. Indeed, *S*. *epidermidis* infections are regarded as prototypic biofilm-associated infections [11]. A key feature characterizing *S*. *epidermidis* biofilms is production of a bacterial derived extracellular matrix, which by embedding the bacterial cells, essentially mediates intercellular adhesion and thereby facilitates stability of the biofilm consortium against mechanical stress [12,13]. In addition, the highly hydrated, mesh work-like character of the biofilm matrix provides a constant extracellular milieu and nutrients to the bacteria allowing for survival even under harsh environmental conditions [13,14].

Chemical analysis of crude *S*. *epidermidis* matrix preparations produced evidence that the biofilm matrix is a heterogeneous mixture of various surface macromolecules including polysaccharides, proteins, teichoic acids and extracellular DNA (eDNA) [15–19]. Given its sticky, glue-like character it was anticipated that these matrix components specifically act as dedicated intercellular adhesins, and significant effort has been placed on characterizing the involved structures and their molecular details [20]. Polysaccharide intercellular adhesin (PIA) ranks among the first molecules for which a specific role in *S*. *epidermidis* biofilm formation was shown. PIA is a homoglycan composed of β-1,6-linked N-acetylglucosaminyl residues synthesized by *icaADBC-*encoded proteins [15,21]. The widespread distribution of *icaADBC* in clinical *S*. *epidermidis* isolates [22–24], and the impaired virulence of an *icaA* mutant 1457-M10 compared to the biofilm-positive wild type in a mouse foreign body infection model demonstrated an important role of PIA for *S*. *epidermidis* pathogenicity [9,25]. In addition to PIA, more recently specific proteins with intercellular adhesive properties have been described. Most notably, the giant 1 MDa extracellular matrix binding protein Embp has been identified to govern cell aggregation and biofilm accumulation [17,26]. In addition, the 140 kDa Accumulation associated protein (Aap) forms localized tufts or fibers on the *S*. *epidermidis* cell surface and contributes to bacterial aggregation [16,27]. Bioinformatic analysis showed that Aap consists of an N-terminal YSIRK-motif containing export signal, a C-terminal LPXTG-motif essential for covalent binding of the protein to the cell wall, and two major domains A and B [28,29]. While structure and specific function of domain-A is largely unknown, the structure of domain-B was resolved more recently, providing evidence for an elongated molecular architecture composed of 128 amino acid repeats (G5 or β-triple helix (TH)-β domains) [28,30]. Aap is subject to proteolytic processing, resulting in expression of at least two Aap species, either full length protein or a repetitive domain-B that results from cleavage at the intersection (amino acid 596) between domain A and B [16]. Proteolytic cleavage is necessary for Aap-mediated biofilm accumulation, and domain-B carries the intercellular adhesive properties of Aap that promote the biofilm phenotype [16].

Importantly, analysis of *S*. *epidermidis* strains that form PIA, Embp or Aap-dependent biofilms exhibited a differential spatial distribution of intercellular adhesins [12]. While PIA and Embp were abundantly found on the bacterial surface and within the intercellular space, Aap exhibited a strict surface localization and was not present within the matrix. These differences clearly suggest that intercellular adhesins use variable mechanisms to stabilize the bacterial biofilm. Indeed, PIA, Aap and Embp can functionally substitute for each other and independently mediate cell aggregation and biofilm accumulation [12,16,17,26]. Nevertheless, it is clear that in order to function as an intercellular adhesin, biofilm matrix components like PIA, Aap, or Embp all have to interact with bacterial cell surface structures and *vice versa*. While these structures are essential for recruitment of bacteria to and stabilization within the biofilm consortium, there is only limited knowledge on the nature of these important interactions and the potentially involved interaction partners. The central aim of this study therefore was to test the hypothesis that *S*. *epidermidis* biofilm formation involves specific interactions between cell wall and matrix components and to analyze how these interactions could contribute to establishment and maintenance of surface adherent multilayered *S*. *epidermidis* cell consortia.

## Results

### Identification of Aap domain-B interaction partners

**Fig 1:**
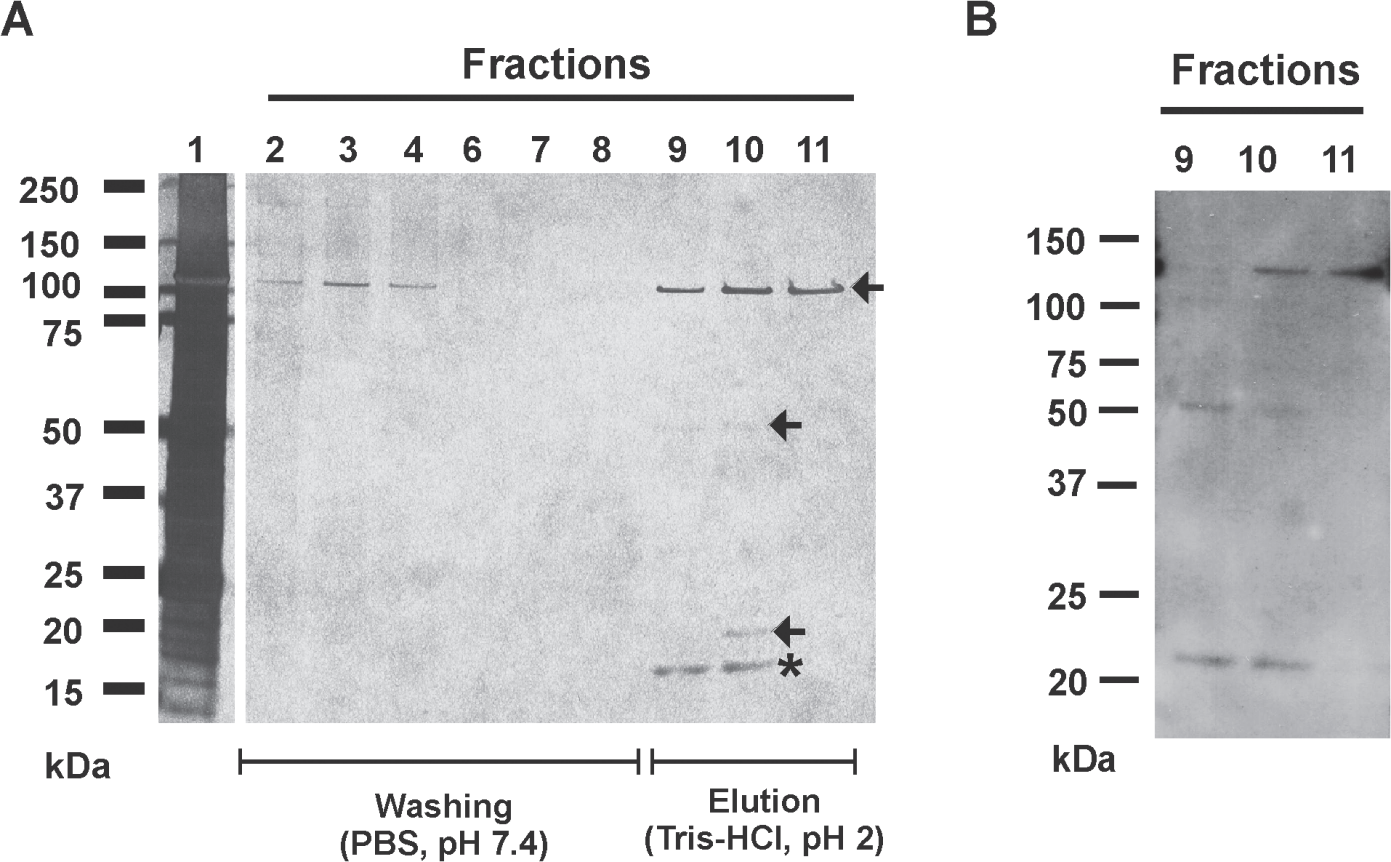
Affinity purification of Aap domain-B interaction partners from crude biofilm matrix preparations. **(A)** SDS-PAGE analysis of fractions collected from affinity chromatography in which recombinant Aap domain-B was coupled to an NHS-activated sepharose column (GE Life Sciences, Freiburg, Germany). Lane 1 shows the input protein preparation that was loaded onto the column. Lanes 2–8: fractions collected during washing with PBS (pH 7.4). Lanes 9–11: fractions collected during elution with Tris-HCl. Proteins were made visible by silver staining (Pierce silver stain kit). Mass spectrometry identified proteins from lanes 9–11 as AtlE (arrows) and hypothetical protein YP_187866 (star). **(B)** Ligand binding assay showing binding of rDomain-B to proteins eluted during affinity purification. Fractions 9–11 were separated by SDS-PAGE and blotted onto a PVDF membrane. After blocking the membrane was incubated with biotinylated rDomain-B (5 μg/ml) and bound ligand was detected by peroxidase-coupled streptavidin and chemiluminescence.

Following the hypothesis that during biofilm accumulation, *S*. *epidermidis* cell wall structures interact with components of the biofilm matrix, affinity chromatography was performed in order to enrich and identify biofilm matrix proteins with Aap domain-B binding activity. To this end, concentrated crude biofilm matrix preparations from *S*. *epidermidis* 1457 were loaded onto NHS-Sepharose columns to which recombinantly expressed domain-B (rDomain-B) was coupled. After extensive washing with PBS (pH 7.4) to remove proteins that were bound non-specifically to the column material, Tris-HCl (pH 2) was used to elute proteins retained on the column with higher affinities. Analysis of collected fractions by SDS-PAGE and subsequent silver staining demonstrated the presence of proteins with an estimated molecular weight of 140, 45, 25 and 20 kDa, respectively (Fig. 1A). Importantly, when identical biofilm matrix preparations were loaded on NHS-columns either functionalized with recombinantly expressed Aap domain-A (rDomain-A) or untreated, no proteins were eluted under identical experimental chromatographic conditions (S1 Fig.).

To further test if the eluted proteins indeed possess rDomain-B binding activity, fractions containing the eluted proteins were used in Far Western Ligand binding assays using biotinylated rDomain-B as a soluble ligand. Here, binding of rDomain-B to all eluted proteins was found (Fig. 1B), giving additional evidence that proteins identified in affinity purification indeed exhibit Aap domain-B binding activity.

To identify candidate Aap domain-B binding proteins electrospray ionisation mass spectrometry (ESI-MS) was applied. Peptides derived from the 110, 45, and 25 kDa proteins after tryptic digestion were unambiguously assigned to *S*. *epidermidis* major Autolysin AtlE (accession number AAW53968). In contrast, peptides derived from the 20 kDa band were mapped to the sequence of a so far uncharacterized, hypothetical *S*. *epidermidis* protein (accession number YP_187866).

### Characterization of hypothetical protein YP_187866

**Fig 2:**
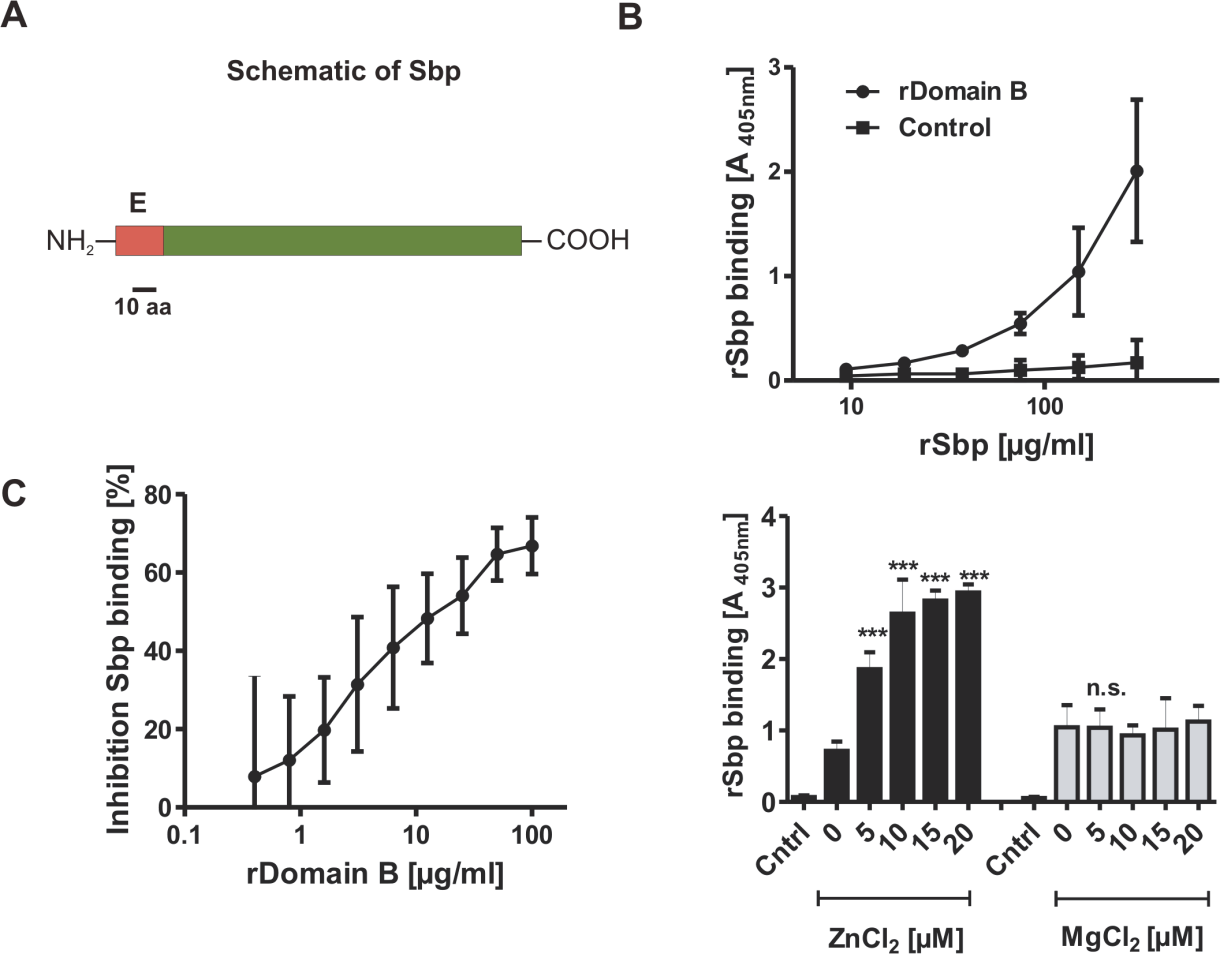
General features of Small basic protein (Sbp) and Biochemical analysis of rDomain-B—Sbp interactions. **(A)** Schematic representation of Sbp. The protein with an anticipated MW of 18 kDa is Encoded by a 513 nt ORF. Bioinformatic analysis identified an N-terminal export signal (E; aa 1–20) but no additional conserved domains. **(B)** Binding of rSbp to rDomain-B immobilized on wells of a 96-well microtiter plate. Increasing amounts of rSbp were allowed to bind to immobilized rDomain-B and bound rSbp was detected by rabbit anti-rSbp antiserum and a alkaline phosphatase coupled goat ant-rabbit IgG. Absorbance at 405 nM is taken as a function of rSbp binding. The untreated (native) polystyrene surface served as a control. Each data point represents mean of 4 values obtained in two independent experiments. Error bars indicate standard deviation. **(C)** Competitive inhibition of rSbp binding to immobilized rDomain-B. rSbp (150 μg/ml) was incubated with increasing amounts of rDomain-B as indicated. After one hour, binding of rSbp to immobilized rDomain-B was tested as described above. Inhibition of rSbp binding was estimated using the formula (1-A_405_ rSbp w/ rDomain-B / A_405_ rSbp w/o rDomain-B) x 100. Each data point represent mean of four values obtained in two independent experiments. Error bars indicate standard deviation. **(D)** Effect of divalent cations on rSbp–rDomain-B interactions. rSbp was allowed to bind to immobilized rDomain-B in the absence or presence of ZnCl_2_ and MgCl_2_, respectively. The unmodified polystyrene surface served as a control (Cntrl). Bound rSbp was detected as described above. Columns represent mean of four values obtained in two independent experiments. Error bars indicate standard deviation. Significant binding differences in the presence of ZnCl_2_ or MgCl_2_ as compared to the control without additional salts (p<0.05, one-way ANOVA with Dunnett’s correction for multiple testing) are indicated (***, p<0.001). n.s., not significant.

Hypothetical 18 kDa protein YP_187866 is encoded by a 513 nucleotide open reading frame SERP0270. Due to its size and basic pI (9.8) we refer to the protein as *S*mall *b*asic *p*rotein (Sbp). Bioinformatic analysis of the deduced amino acid sequence found an N-terminal export signal with a putative cleavage site between amino acids 28 and 29 (http://www.cbs.dtu.dk/services/SignalP/), but no additional conserved motifs, such as a covalent cell wall linkage (e.g. a LPXTG motif) were identified (http://prosite.expasy.org/) (Fig. 2A). Aiming at validating and characterizing Aap domain-B–Sbp interactions on a biochemical level, recombinant rSbp was used as a ligand in solid phase binding assays in which rDomain-B was immobilized to a polystyrene surface (Fig. 2B). Here, a dose dependent binding of rSbp to immobilized rDomain-B was recorded. Specificity of the rSbp–rDomain-B binding was validated in competition experiments where binding of rSbp was dose-dependently inhibited by pre-incubation of rSbp with soluble rDomain-B up to 80% (Fig. 2C). Mounting evidence suggests that Aap domain-B is a zinc-binding protein and Zn^2+^ is necessary for self association of Aap domain-B [28,30]. Therefore the hypothesis was put forward that zinc could also impact Aap domain-B—Sbp interactions. To test this hypothesis, binding of soluble rSbp (150 μg/ml) to immobilized rDomain-B was analyzed in a solid phase assay in the presence of various concentrations of ZnCl_2_ (Fig. 2D). Indeed, while binding of rSbp occurred in the absence of zinc, there was a clear, dose-dependent increase in rSbp binding when ZnCl_2_ was added to the buffer. The maximum binding was reached at 10 μM ZnCl_2_, corresponding to Zn^2+^ concentrations in human plasma. Importantly, addition of MgCl_2_ had no impact on rSbp–rDomain-B interactions, suggesting that augmented binding is not a general effect of divalent cations (Fig. 2D). In summary, these findings establish biochemical evidence for direct Sbp–Aap domain-B interactions.

**Fig 3:**
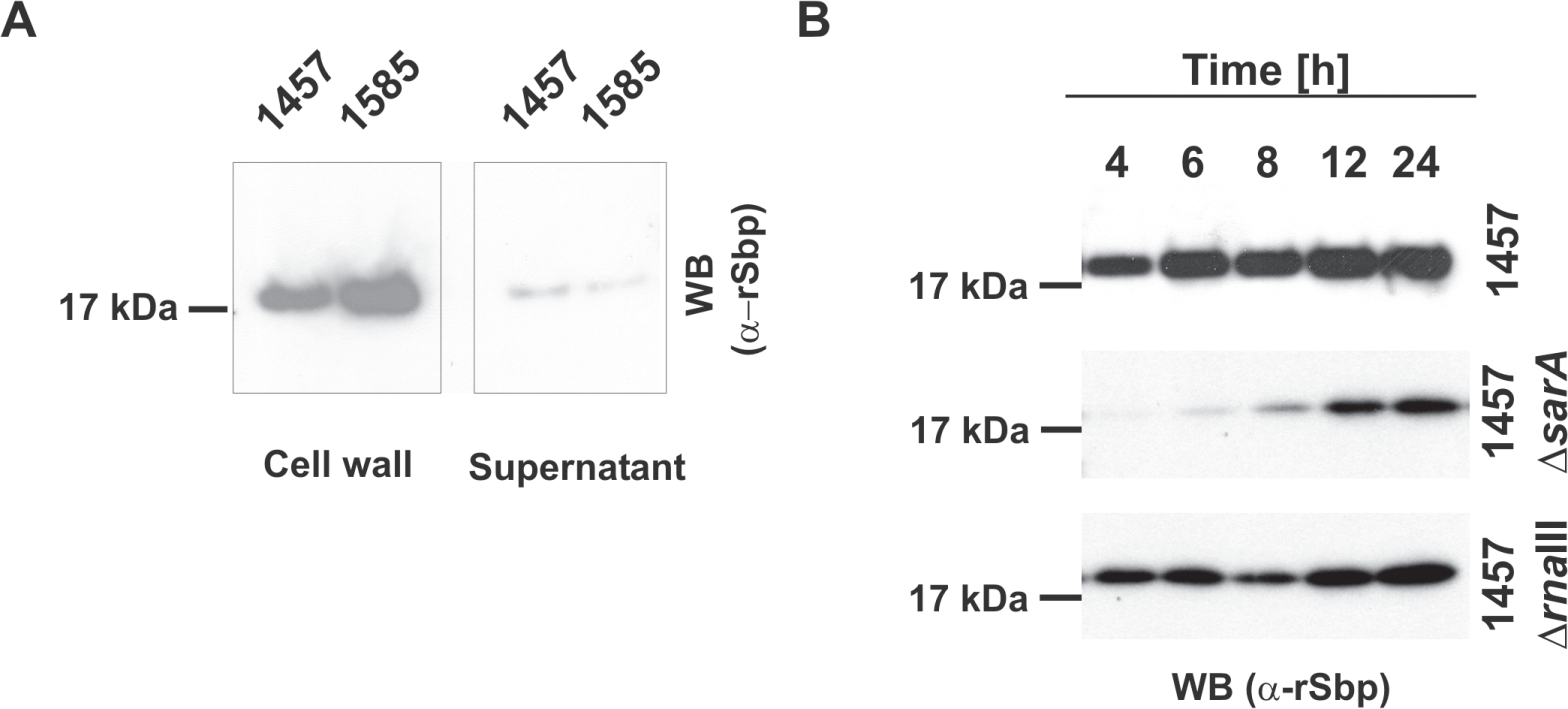
Spatial distribution of Sbp in *S*. *epidermidis* cultures. **(A)** Preparations of cell wall associated proteins and 10-fold concentrated supernatants from *S*. *epidermidis* 1457 (biofilm-positive) and 1585 (biofilm-negative) after static overnight growth were separated by SDS PAGE and blotted onto PVDF-membranes. Sbp was detected after incubation with rabbit anti-rSbp antiserum and anti-rabbit IgG coupled to peroxidase by chemiluminescence. **(B)** Growth phase dependent regulation of Sbp. Cell wall associated proteins were prepared from *S*. *epidermidis* 1457, 1457Δ*sarA*, and 1457Δ*rna*III at different time points during adherent growth in TSB. At each time point cell numbers were adjusted to an identical A_600_ before cell surface associated proteins were isolated by boiling in LDS buffer. After separation of surface associated proteins by SDS-PAGE and blotting onto a PVDF membrane Sbp was detected by chemiluminescence using a rabbit anti-rSbp antiserum and a peroxidase-coupled anti-rabbit IgG. SDS-PAGE analysis proved loading of gels with similar total protein amounts (S2 Fig.).

The identified export signal suggested an extracellular localization of Sbp. Indeed, by using a polyclonal rabbit antiserum raised against recombinant Sbp (rSbp) the protein was detected in preparations of cell wall associated proteins from biofilm-positive *S*. *epidermidis* 1457 and also biofilm-negative *S*. *epidermidis* 1585 (Fig. 3A). Importantly, only small Sbp amounts were found in culture supernatants even after 10-fold concentration (Fig. 3A). Thus, Sbp obviously is an extracellular protein which, after export, is recruited to and accumulates on the bacterial cell surface, while only minor amounts are shed into the supernatant. Sbp was detected in almost identical amounts in protein preparations of sessile bacteria obtained after 4, 6, 8, 12 and 24 hours of growth and normalized for cell densities, demonstrating an expression without significant growth phase dependency (Fig. 3B). In order to get first insights into the integration of Sbp production into superimposed gene regulatory networks, time-dependent Sbp production was also tested in deletion mutants of *S*. *epidermidis* 1457 defective in global gene regulators *sarA* and *agr* (1457Δ*sarA* and 1457Δ*rnaIII*, respectively). While inactivation of *rnaIII* had no impact on Sbp production, inactivation of *sarA* abolished or severely impaired quantitative Sbp expression at 4, 6, and 8 hours of growth (Fig. 3B). Thus, Sbp production during exponential and early stationary growth phase clearly depends on SarA.

PCR analysis confirmed the presence of *sbp* in 52/52 (100%) invasive *S*. *epidermidis* strains isolated from prosthetic joint infections (described in [23]) as well as in 40/40 (100%) colonizing *S*. *epidermidis* strains isolated from nose swabs (described in [22]). Orthologues of Sbp sharing sequence similarity with the *S*. *epidermidis* protein are present in other coagulase-negative staphylococcal species (e.g. *S*. *caprae*, accession number WP_002452548.1: amino acid identity 76.9%, similarity 98.8%; *S*. *lugdunensis*, accession number WP_002459857.1: amino acid identity 63.3%, similarity 93.5%) and *S*. *aureus* (e.g. strain Newman, accession number YP_001331619.1; amino acid identity 60%, similarity 92.8%).

### Spatial distribution of Sbp in *S*. *epidermidis* biofilms

**Fig 4:**
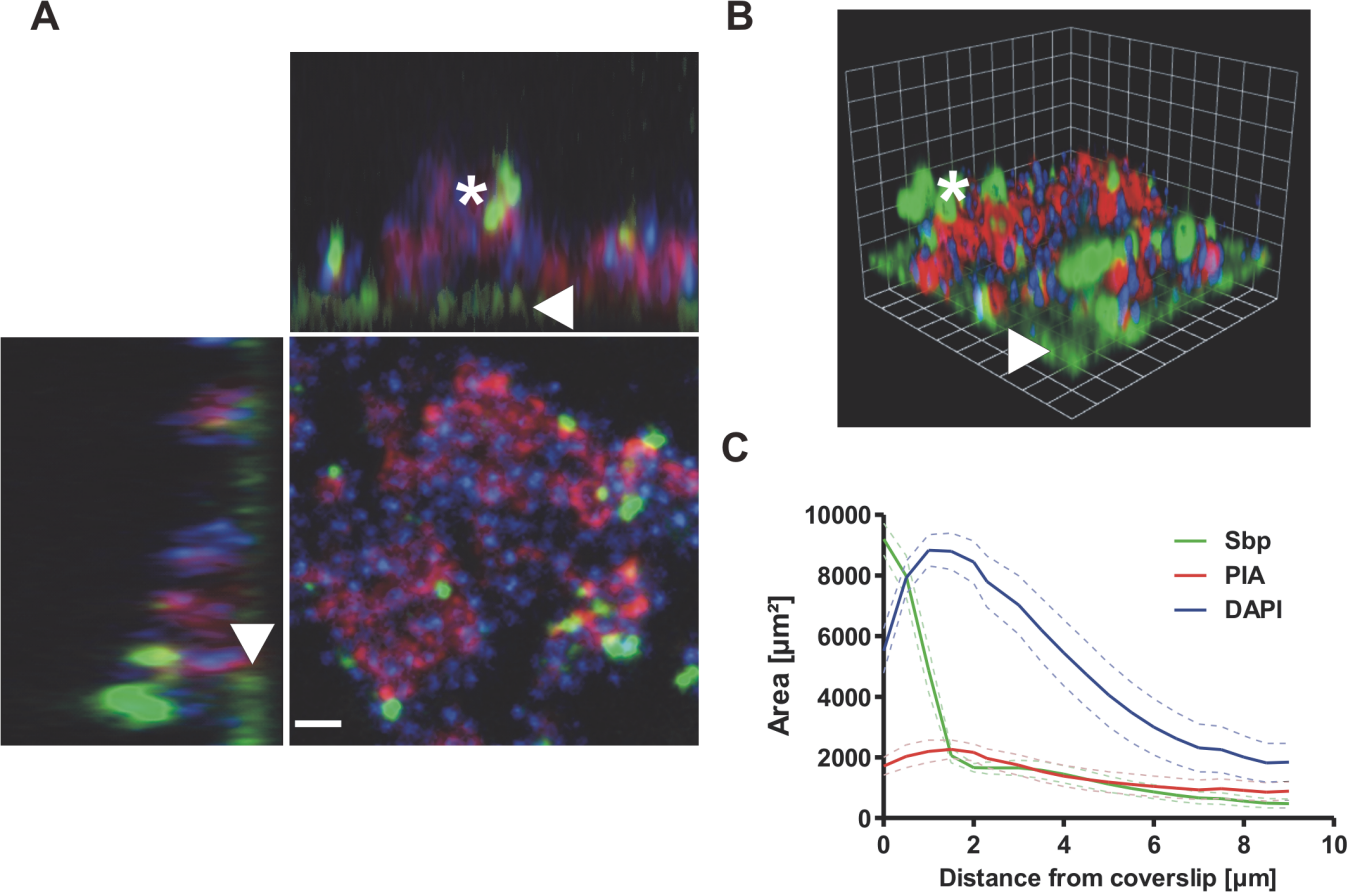
Distribution of Sbp in living biofilms. Confocal laser scanning microscopy images of *S*. *epidermidis* 1457 biofilms grown overnight under static conditions. Bacteria were stained using DAPI (blue), while PIA and Sbp were detected by WGA coupled to Alexa 568 (red) and rabbit anti-rSbp / anti-rabbit IgG-Alexa488 (green), respectively. **(A, B)** Confocal images of DAPI-labelled *S*. *epidermidis* 1457 (blue), PIA (red) and Sbp (green). Two dimensional projections along the XY / XZ-axes (A) and a three dimensional reconstruction (B) clearly show a preferential localization of Sbp at the biofilm—substrate interface (*arrowheads*) but also within higher parts of the biofilm (*asterisk*) where the protein partially co-localizes with PIA. Scale bar = 2 μm (A), grid unit = 1.76 μm (B). **(C)** Quantitative analyses of the image by planimetry shows the distribution of the labelled components along the z-axis in a 109x109μm^2^ field of view. The majority of Sbp localizes near the coverslip, whereas PIA is found throughout all layers of the biofilm. 10 images were analyzed and the measured area was plotted as mean +/- SEM.

Having shown the preferential surface localization of Sbp, next a set of experiments was performed to characterize the presence and spatial distribution of Sbp in living biofilms. To this end, statically grown biofilms of *S*. *epidermidis* 1457 were investigated by CLSM. While WGA-Alexa568 was used to stain for the principal biofilm matrix component PIA, Sbp was detected by rabbit anti-rSbp antiserum and anti-rabbit IgG coupled to Cy5. After 22 hours of growth, Sbp was detected in association with *S*. *epidermidis* cells, but predominantly localized to biofilm matrix (Fig. 4A). In contrast to PIA, an important biofilm matrix component, detailed analysis showed that Sbp significantly differed in its spatial matrix allocation. PIA, although not evenly distributed in the horizontal dimension, was detected throughout all layers of the biofilm, forming a partially compact, partially net-like extracellular matrix embedding the bacteria (Fig. 4A). In sharp contrast, detection of Sbp by immunofluorescence demonstrated a significant spatial heterogeneity of the protein: Sbp accumulated in huge humps or clusters unevenly interspersed within the biofilm architecture, but more strikingly, Sbp was concentrated at the biofilm—surface interface. Here, the protein formed a continuous, film-like structure (Fig. 4B). To objectively assess the differential distribution of PIA and Sbp, the fluorescence intensities in each transverse section were measured and plotted as a function of biofilm depth (Fig. 4C). These measurements confirmed that Sbp, though present in all levels of the biofilm, preferentially accumulated at the biofilm–surface interface. In contrast, PIA evenly localized to all levels of the biofilm (Fig. 4C).

### Functional role of Sbp in *S*. *epidermidis*–surface interactions

**Fig 5:**
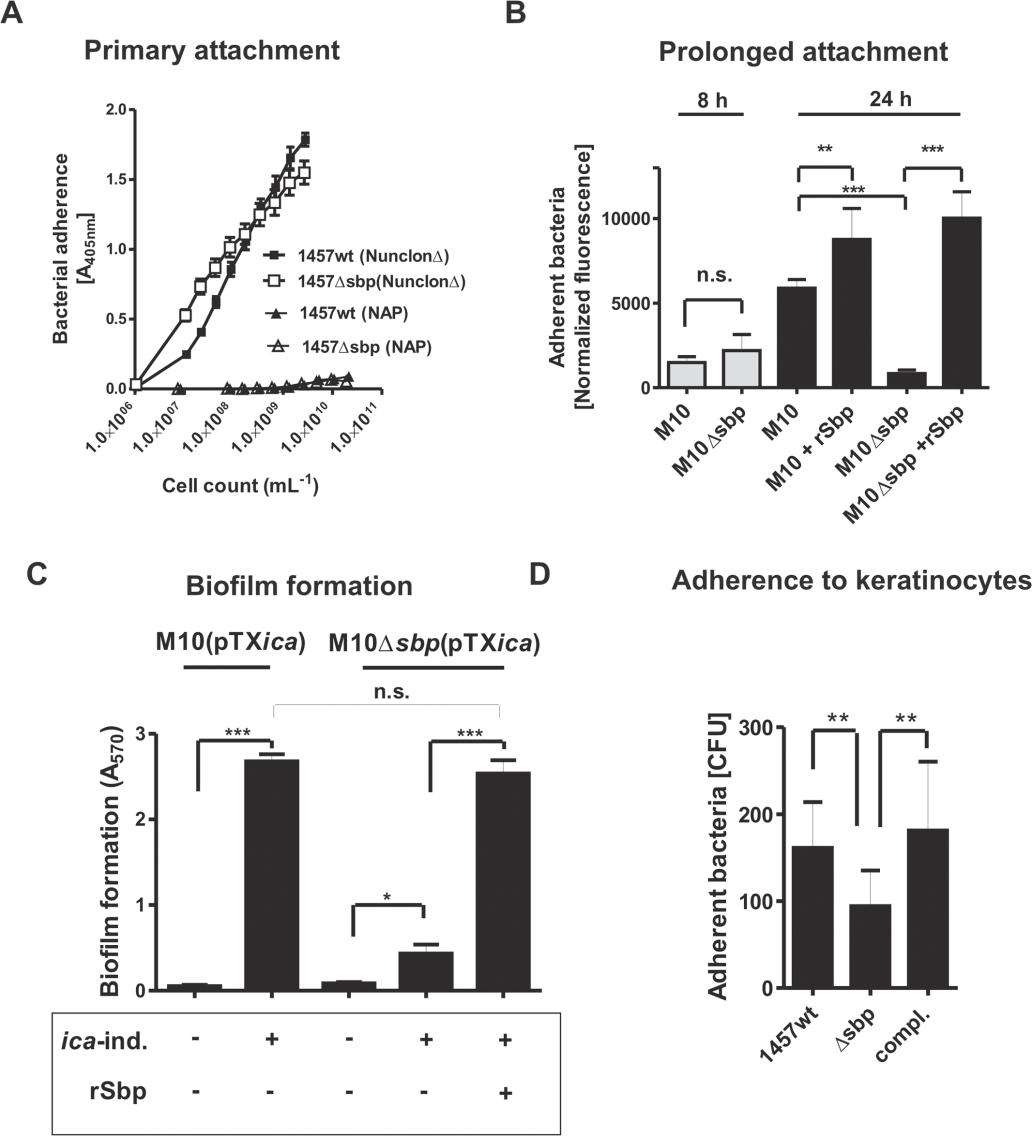
Functional role of Sbp in primary attachment. **(A)** Quantification of surface bound bacteria on pro-adherent polystyrene after one hour (rapid adherence). Binding of 1457 and 1457Δ*sbp* to cell culture treated polystyrene (NunclonΔ, Roskilde, Denmark) and non-adhesive polystyrene (NAP; Greiner, Frickenhausen, Germany). Each data point represents the mean A_405_ value derived from 18 replicate measurements obtained in two independent experiments. Error bars indicate standard deviation. **(B)** Prolonged *S*. *epidermidis* adherence to unmodified and rSbp-coated non-adhesive polystyrene (NAP). Gfp-expressing 1457-M10(pGFP) and 1457-M10Δ*sbp*(pGFP) were grown in TSB under static conditions for 8 and 24 hours. After washing adherent cells were quantified by determining fluorescence intensity (excitation 485 nm, emission 535 nm). Columns represent normalized fluorescence values (i.e. fluorescence intensities normalized against respective bacterial cell densities). Error bars indicate standard deviation. Significant differences (p<0.05; one-way ANOVA with Bonferroni’s correction for multiple testing) are indicated by stars (**, p<0.01; ***, p<0.001). n.s., not significant. **(C)** Biofilm accumulation on non-adhesive polystyrene (NAP). 1457-M10(pTX*icaADBC*) and 1457-M10Δ*sbp*(pTX*icaADBC*) were grown under static conditions for 24 hours in the presence or absence of 3% [wt/vol] xylose. After washing, adherent cells were stained with Gentiana violet and biofilm formation was quantified at 570 nm. Biofilm formation of 1457-M10Δ*sbp*(pTX*icaADBC*) under inducing was also tested after coating of NAP surfaces with rSbp. Columns represent means of 6 values obtained in 3 independent experiments. 1457-M10 and 1457-M10Δ*sbp* complemented with plasmid pTX*icaADBC* (allowing for inducible PIA production) was used here to avoid confounding effects of *sbp* inactivation on PIA production (as demonstrated in Fig. 8A). Error bars indicate standard deviation. Significant differences (p<0.05; one-way ANOVA with Bonferroni’s correction for multiple testing) are indicated by stars (*, p<0.05; ***, p<0.001). n.s., not significant. **(D)** Adhesion of *S*. *epidermidis* strain 1457, 1457Δ*sbp* and the complemented mutant to HaCaT keratinocytes. Columns represent mean of 10 values obtained in 5 independent experiments, error bars depict standard deviation. Differences between 1457 and 1457Δ*sbp* as well as 1457Δ*sbp* and the complemented mutant were significant different (p<0.01, Wilcoxon rank sum test).

Interactions with abiotic surfaces are pivotal for the initial adherence phase of bacterial biofilm formation, but are also crucial for robust surface retention of aggregated *S*. *epidermidis* cell clusters during subsequent biofilm accumulation. Building on the preferential detection of Sbp at the biofilm—surface interface we speculated that *S*. *epidermidis* can use Sbp to prime surfaces for robust bacterial adherence. To test this hypothesis *S*. *epidermidis* adherence was analyzed using polystyrene micro titer plates (Greiner, Frickenhausen, Germany) possessing only a minimal inherent bacterial binding capacity (referred to as *N*on-*a*dhesive *p*olystyrene [NAP]) [17]. As expected, after one hour neither the 1457 wt nor the 1457Δ*sbp* mutant displayed a significant adherence to this type of surface (Fig. 5A). Importantly, to ensure measurement of adherent bacteria without relevant confounding by bacterial cell cluster formation, a PIA- and aggregation-negative *icaA*::Tn*917* mutant, 1457-M10, and its isogenic *sbp* mutant 1457-M10Δ*sbp* were used in all experiments, and detection of Gfp-emitted fluorescence was used for quantification of adherent cells.

To test if endogenous Sbp fosters sustained bacterial adherence to NAP, surface adherent 1457-M10(pGFP) and Sbp-negative mutant 1457-M10Δ*sbp*(pGFP) were quantified after 24 hours of growth and subsequent rigorous washing. Indeed, despite reaching identical cell densities (as determined by assessment of optical densities at 600 nm after 24 h), assessment of fluorescence- intensities after washing indicated that significantly more (p<0.05, Mann-Whitney test) 1457-M10(pGFP) cells (mean surface adherent CFU = 1.4*10^7^) remained on the surface compared to 1457-M10Δ*sbp*(pGFP) (mean surface adherent CFU = 8.5*10^5^). Thus, Sbp-producing strain 1457-M10(pGFP) more stably attached to NAP-surface compared to Sbp-negative 1457-M10Δ*sbp*(pGFP) (Fig. 5B). Immobilization of rSbp on NAP not only restored the adherence defect of 1457-M10Δ*sbp*(pGFP), but also resulted in increased adherent cell numbers of 1457-M10(pGFP) (mean surface adherent CFU = 2.2*10^7^) (Fig. 5B). Importantly, after eight hours of incubation of 1457-M10 no significant differences in adherent cell numbers were noted between 1457-M10(pGFP) and 1457-M10Δ*sbp*(pGFP) (Fig. 5B). Though Sbp was already detectable on the surface at this time point, a significant increase of Sbp abundance was detected after 24 hours of incubation (S3 Fig.). Thus, it appears that a critical Sbp threshold quantity exists that determines the pro-adherent effect of Sbp on *S*. *epidermidis* surface retention. Taken together these findings strongly suggest that Sbp, while not being necessary for primary attachment to NAP during the very early phase of surface colonization, its deposition at the bacterium–substrate interface during growth is functionally important for establishment of sustained, mechanically robust tethering of *S*. *epidermidis* to an abiotic surface at later stages of sessile life styles.

We next asked if Sbp-mediated binding of *S*. *epidermidis* to NAP is also necessary for surface retention of a mature biofilm consortium. To this end, biofilm formation of Sbp-producing or—negative strains was tested. Since inactivation of *sbp* resulted in reduced PIA levels in *S*. *epidermidis* 1457 (see below for detailed analysis), in these experiments 1457-M10(pTX*icaADBC*) and 1457-M10Δ*sbp*(pTX*icaADBC*) were used, allowing for inducible *icaADBC*- expression and PIA- synthesis (Table 1). PIA-producing strain 1457-M10(pTX*icaADBC*) established a strong surface adherent biofilm resistant against rigorous washing procedures. In contrast, cell aggregates of PIA-positive but Sbp-negative 1457-M10Δ*sbp*(pTX*icaADBC*) were easily washed away, resulting in a biofilm-negative phenotype. Only by rescuing the adherence defect through immobilization of rSbp on the NAP surface, 1457-M10Δ*sbp*(pTX*icaADBC*) biofilms were able to withstand mechanical stress and to remain on the surface (Fig. 5C).

**Table 1:** Strains used in the study.

Strain	Characteristics	Reference
1457	*icaADBC*-positive, *aap*-positive, strong biofilm formation.	[43]
1457(pGFP)	In trans *gfp* expression from plasmid pCM29.	[57]
1457Δ*sarA*	Mutant carrying a deletion of master regulator *sarA*; Tet^R^.	[58]
1457Δ*rna*III	Mutant carrying a deletion of *rna*III, major effector of the *agr* regulatory cascade; Tmp^R^.	[59]
1457Δ*aap*	Aap-negative knock-out mutant; Tet^R^.	[48]
1457Δ*sbp*	Markerless *sbp* knock-out mutant.	This study
1457Δ*aap*Δ*sbp*	Aap- / Sbp-negative mutant, obtained by phage transduction of *aap*::*tetM* into 1457Δ*sbp*; Tet^R^.	This study
1457Δ*sbp*(pRB*sbp*)	Complemented mutant of 1457Δ*sbp*; *in trans* expression of *sbp* from its natural promoter [47]; Cm^R^.	This study
1457Δ*aap*Δ*sbp*(pRB*sbp*)	Complemented mutant of 1457Δ*aap*Δ*sbp*; in trans expression of *sbp;* Tet^R^, Cm^R^.	This study
1457Δ*aap*Δ*sbp*(pRBDomain-B)*	Complemented mutant of 1457Δ*aap*Δ*sbp*; constitutive in trans expression of *aap* domain-B (aa 596–1507); Tet^R^, Cm^R^.	This study [16]
1457-M10	*icaA*::Tn*917* insertion mutant, PIA- and biofilm-negative.	[32]
1457-M10Δ*aap*	Aap-negative mutant of 1457-M10 obtained by phage transduction of *aap*::*tetM*; Tet^R^, Ery^R^.	This study
1457-M10Δ*sbp*	Markerless *sbp* knock-out mutant; Ery^R^.	This study
1457-M10Δ*aap*Δ*sbp*	Aap- / Sbp-negative mutant obtained by phage transduction of *aap*::*tetM* into 1457-M10Δ*sbp*; Tet^R^, Ery^R^.	This study
1457-M10Δ*aap*(pRBDomain-B) *	Complemented mutant 1457-M10Δ*aap*; constitutive *in trans* expression of Aap domain-B (aa 596–1507); Tet^R^, Ery^R^, Cm^R^.	This study [16]
1457-M10Δ*aap*Δ*sbp*(pRB*sbp*)	Complemented mutant 1457-M10Δ*aap*Δ*sbp*; *in trans* expression of *sbp*; Tet^R^, Ery^R^, Cm^R^.	This study
1457-M10Δ*aap*Δ*sbp*(pRBDomain-B) *	Complemented mutant 1457-M10Δ*aap*Δ*sbp*; constitutive *in trans* expression of Aap domain-B(aa 596–1507); Tet^R^, Ery^R^, Cm^R^.	This study
1457-M10*(*pTX*icaADBC)*	Complemented mutant 1457-M10; *in trans* expression of *icaADBC* from a xylose-inducible promoter; Ery^R^, Tet^R^.	[32]
1457-M10Δ*sbp (*pTX*icaADBC)*	Mutant 1457-M10Δ*sbp* complemented with plasmid pTX*icaADBC* allowing for expression of *icaADBC* in an Sbp-negative background; Ery^R^, Tet^R^	This study
1585	Biofilm negative, clinically significant isolate from a port catheter infection; *icaADBC*-negative; *aap*-negative; biofilm-negative	[16,17]
1585Δ*sbp*	Markerless *sbp* knock-out mutant of *S*. *epidermidis* 1585	This study

Cm: Chloramphenicol; Ery: Erythromycin; Tet: Tetracycline; TMP: trimethoprim

*Plasmid pRBDomain-B was originally referred to pRB*aap*R_T_ [16]

In order to test the idea that Sbp could also play a role during *S*. *epidermidis* colonization of biotic, e.g. epidermal surfaces, we also tested adherence of *S*. *epidermidis* 1457, 1457Δ*sbp* and the complemented mutant to human keratinocytes. To this end, bacteria were allowed to interact with confluent layers of HaCaT cells and adherent cells were enumerated. Inactivation of *sbp* induced a significant reduction of adherent *S*. *epidermidis* cells, while complementation restored adherent capacities to wild type levels (Fig. 5D). Reduced binding to keratinocytes after inactivation of *sbp* was also observed in the background of *icaADBC*-negative *S*. *epidermidis* 1585 and thus, is unrelated to potential changes in PIA-production resulting from abolished Sbp production (S4 Fig.).

### Functional role of Sbp in *S*. *epidermidis* biofilm accumulation

**Fig 6:**
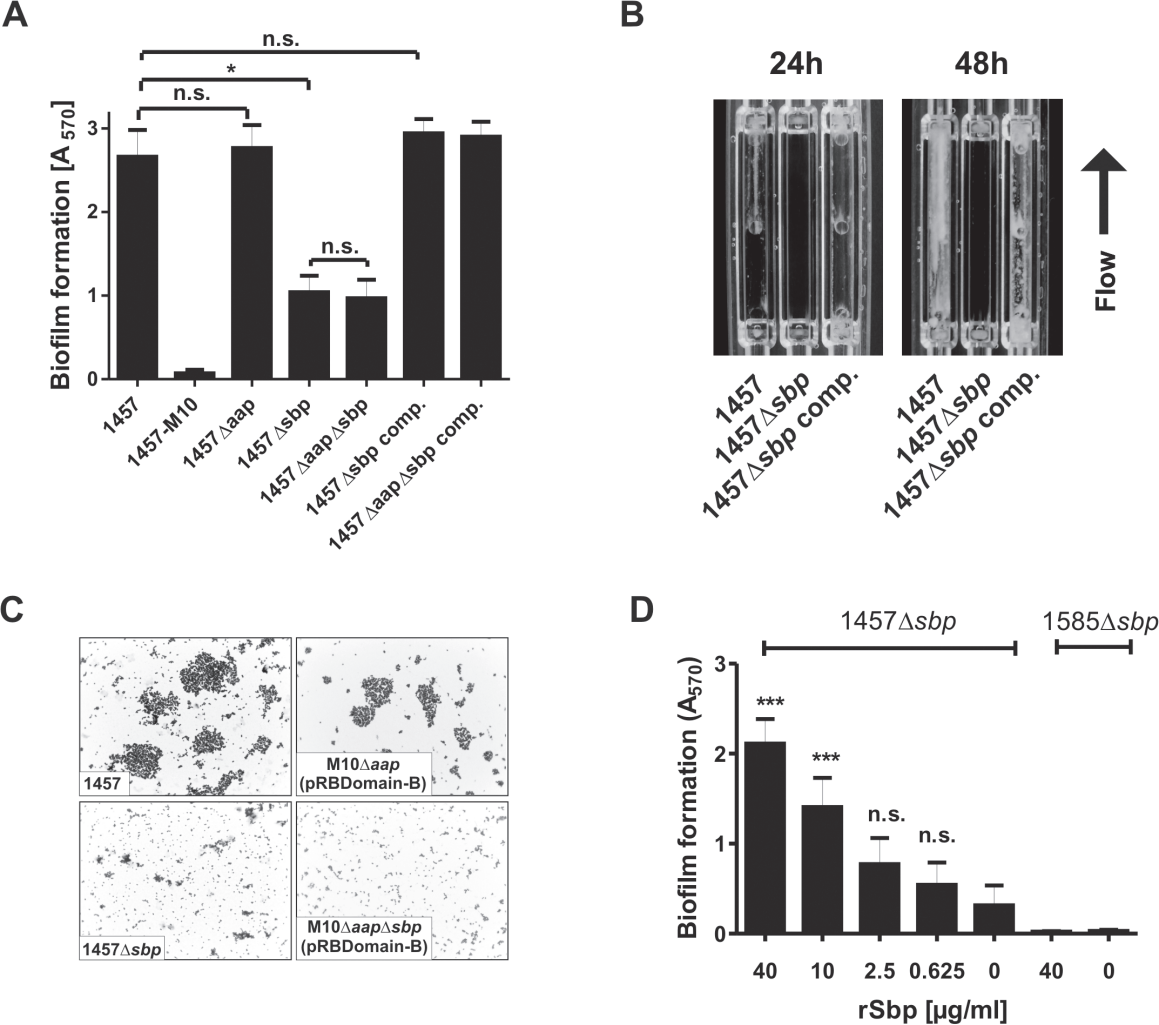
Functional role of Sbp in *S*. *epidermidis* 1457 biofilm formation. **(A)** Photometric quantification of biofilm formation after overnight growth of 1457, 1457Δ*sbp*, 1457Δ*aap*, 1457Δ*aap*Δ*sbp*, 1457Δ*sbp* complemented with pRB*sbp* and 1457Δ*aap*Δ*sbp* complemented with pRB*sbp*. 1457-M10 (PIA-negative) served as a control. Adherent cells were stained with gentiana violet before absorption at 570 nm was assessed. Columns represent means of twelve values obtained in three independent experiments. Error bars indicate standard deviations. Significant differences (Kruskall-Wallis one-way ANOVA with Dunn’s multiple comparison test) are indicated (*, p<0.05). n.s. not significant. **(B)** Analysis of biofilm formation under flow conditions. *S*. *epidermidis* 1457, 1457Δ*sbp* and the complemented mutant were grown in TSB + 0.5% glucose for 48 h at a flow rate of 1 ml/min. **(C)** Microscopic analysis of *S*. *epidermidis* 1457, 1457Δ*sbp*, 1457-M10Δ*aap*(pRBDomain-B), and 1457-M10Δ*aap*Δ*sbp*(pRBDomain-B). Cells were scraped from cell culture plates after static over night growth and appropriate dilutions were allowed to dry on glass cover slips. Bacteria were Gram-stained. Images were taken at 1000 x magification. **(D)** Induction of biofilm formation by exogenous recombinant rSbp. Biofilm formation of 1457Δ*sbp* and 1585Δ*sbp* was quantitatively assessed in the presence of varying amounts of purified rSbp. After overnight growth, adherent cells were stained using gentiana violet, and biofilms were quantified spectrophotometrically at 570 nm. Columns represent means of 6 values obtained in three independent experiments. Error bars indicate standard deviation. Significant differences compared to the control (no exogenous rSbp; One-way ANOVA with Dunnett’s correction for multiple testing) are indicated (***, p<0.001). n.s., not significant.

**Fig 7:**
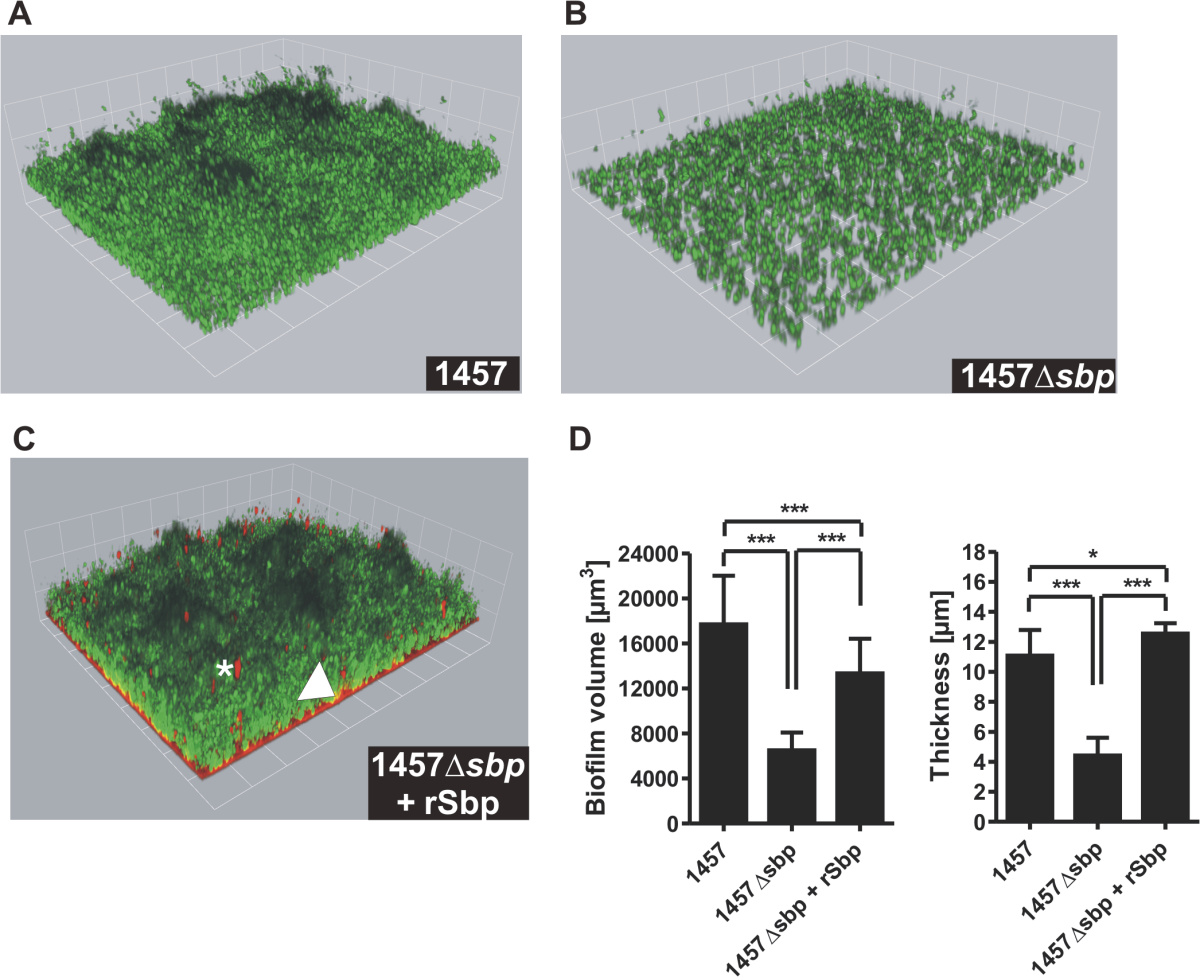
Microscopic analysis of 1457Δ*sbp*. **(A—C)** Three dimensional reconstruction of biofilms from Gfp-expressing *S*. *epidermidis* 1457 (A), 1457Δ*sbp* (B) and 1457Δ*sbp* grown in the presence of fluorescence-labelled rSbp-DyLight550 (1.5 μg/ml; red) (C). The arrowhead indicates localization of rSbp at the biofilm—substrate interface, the asterisk highlights localization of rSbp within the biofilm matrix. A detailed demonstration of rSbp-DyLight550 distribution is shown in S6A Fig.. Fluorescence labelling did not alter the biofilm-inducing properties of rSbp (S6B Fig.). Grid unit = 11.62 μm **(D)** Quantification of mean biofilm volume and thickness. 18 randomly chosen biofilm CLSM images obtained in three independent experiments were analyzed for each strain. Analysis was carried out using the Volocity software package. Error bars depict standard deviations. Significant differences (p<0.05; one-way ANOVA with Bonferroni’s correction for multiple testing) are indicated (*. p<0.05; ***, p<0.001). Differences indicate between wild type 1457 and 1457Δ*sbp* complemented with rSbp indicate the potential importance of the Sbp source for *S*. *epidermidis* biofilm structure. rSbp-DyLight550 fully reconstituted biofilm formation in 1457Δ*sbp* in standard micro titer plate biofilm assays (S6B Fig.).

We next thought to investigate the role of Sbp in *S*. *epidermidis* biofilm accumulation. These assays were carried out using pro-adherent NunclonΔ surfaces, to which *S*. *epidermidis* 1457 and 1457Δ*sbp* bind with similar efficiency (Fig. 5A), thus allowing for assessment of biofilm accumulation defects without confounding effects of attachment differences. In static biofilm assays, inactivation of *sbp* in PIA-producing *S*. *epidermidis* 1457 induced a significant, roughly 60% reduction of biofilm mass (Fig. 6A), and under flow conditions the inability to establish adherent growth was even more pronounced after 24 as well as 48 hours of growth, respectively (Fig. 6B). The severe impact of *sbp* knock-out on biofilm formation was also assessed by CLSM analysis of static biofilms grown for 20 hours, showing a clear reduction of the average biofilm volume and average biofilm thickness in 1457Δ*sbp* compared to the wild type 1457 (Fig. 7A,B,D). Strikingly, in contrast to cluster forming wild type 1457, 1457Δ*sbp* was present predominantly as single cells and established only small cell clusters, giving rise to the assumption that the reduced biofilm forming capacity of 1457Δ*sbp* most likely is linked to a defect in intercellular adhesion (Fig. 6C). Complementation of 1457Δ*sbp* with plasmid pRB*sbp* allowing for *in trans* expression of *sbp* (S5C Fig.) restored the biofilm-positive phenotype in 1457Δ*sbp* (Fig. 6A-B). To test if *S*. *epidermidis* 1457Δ*sbp* can also use exogenous Sbp, purified rSbp was added to growing cultures of 1457Δ*sbp*. This measure induced 1457Δ*sbp* biofilm formation in a dose-dependent manner (Fig. 6D), and the average biofilm volume and thickness returned to wild type levels (Fig. 7C-D). Importantly, at rSbp concentrations similar to that produced by wild type *S*. *epidermidis* 1457 (roughly 1.5 μg/ml), the recombinant protein exhibited a spatial distribution almost identical to endogenously produced Sbp (Figs. 7C, S6A). Taken together, these results demonstrate that the reduced biofilm formation in 1457Δ*sbp* is indeed a consequence of aborted Sbp production which, therefore, is necessary for cell aggregation and biofilm formation in this strain. Conversely, even at high concentrations rSbp did not induce biofilm- formation in *aap*- and *icaADBC*-negative *S*. *epidermidis* 1585Δ*sbp* (Fig. 6D). Taking into account that Sbp is also produced by biofilm-negative *S*. *epidermidis* strains (e.g. *S*. *epidermidis* 1585, Fig. 2), and over-expression of Sbp in surrogate host *S*. *carnosus* TM300 did not induce a biofilm-positive phenotype (S7 Fig.), it appears that Sbp alone is not sufficient for mediating intercellular adhesion, but needs additional partners to induce cell aggregation and biofilm accumulation. Thus, given the importance of PIA in *S*. *epidermidis* 1457 biofilm formation [21], direct or indirect effects of Sbp on PIA-dependent biofilm formation appeared as reasonable explanations for the observed biofilm mass reduction in 1457Δ*sbp*.

### Impact of Sbp on PIA-dependent biofilm formation

**Fig 8:**
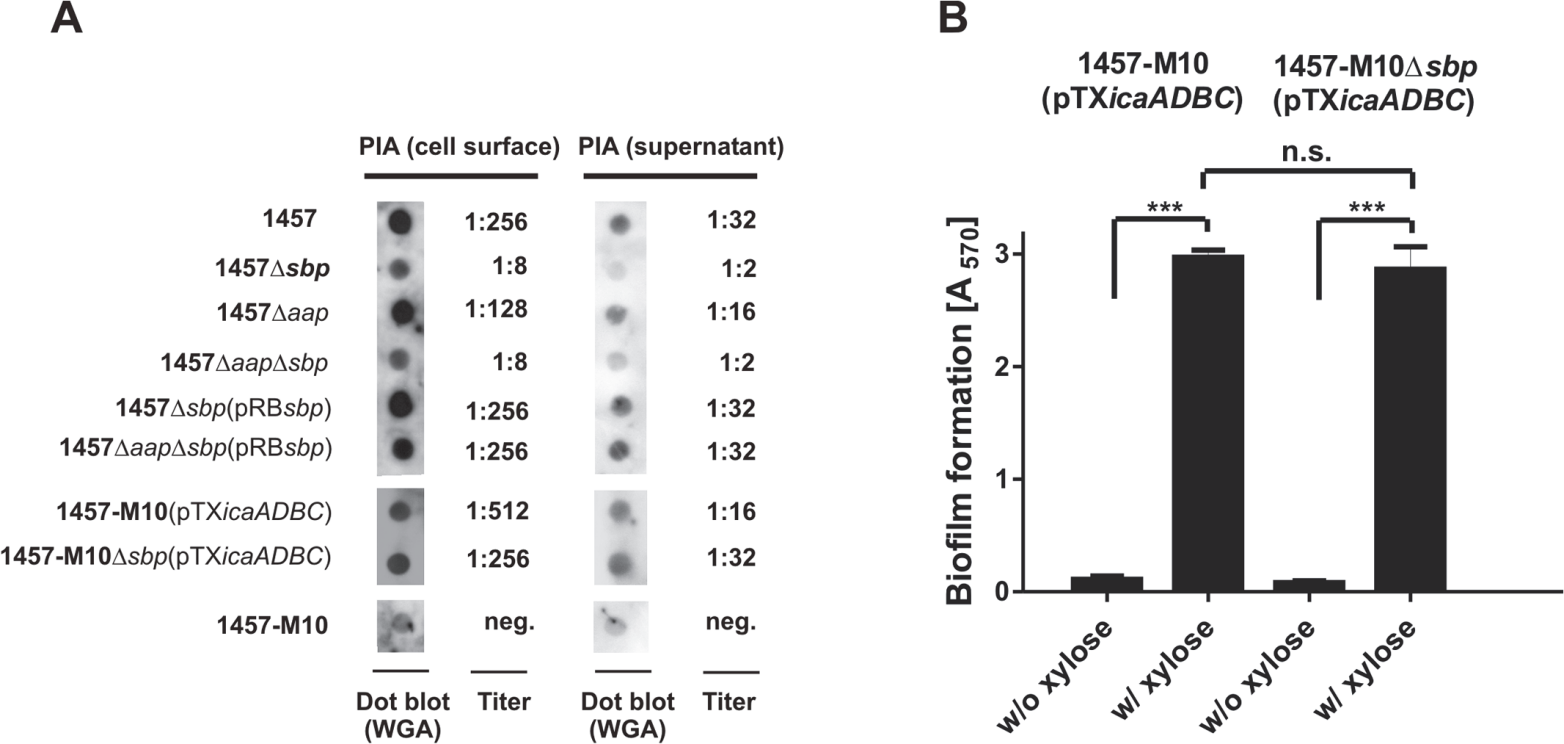
Interconnection between Sbp production and PIA synthesis. **(A)** Quantification of PIA production in *S*. *epidermidis* 1457, 1457Δ*sbp* 1457Δ*aap* and 1457Δ*aap*Δ*sbp* by dot blot analysis. Serial dilutions of cell wall extracts were spotted onto PVDF membranes which were then incubated with WGA coupled to peroxidase. Bound WGA was then visualized by chemiluminescence. PIA titers were defined as the highest dilution giving a signal above the background (as determined by parallel analysis of 1457-M10). Dot blots show results obtained at 1:8 (cell wall preparations) and 1:2 (supernatants) dilution. 1457-M10(pTX*icaADBC*) and 1457-M10Δ*sbp*(pTX*icaADBC*) were grown in the presence of 3% [wt/vol] xylose for induction of *icaADBC* expression. **(B)** Analysis of biofilm formation by 1457-M10 and 1457-M10Δ*sbp* complemented with pTX*icaADBC* allowing for xylose-inducible *in trans* expression of *icaADBC*. Bacteria were grown overnight in TSB or TSB supplemented with xylose (3% [w/v]), respectively. Following washing procedures adherent bacteria were stained with gentiana violet. Columns represent means of 12 values obtained in three independent experiments. Error bars indicate standard deviation. Significant differences (p<0.05; one-way ANOVA with Bonferroni’s correction for multiple testing) are indicated by stars (***, p<0.001). n.s., not significant.

Following the observation of a partial co-localization of Sbp and PIA in living *S*. *epidermidis* 1457 biofilms and the predicted charged character of both molecules, we speculated if impaired biofilm formation of 1457Δ*sbp* indicates a structural involvement of Sbp in PIA recruitment to the bacterial cell surface, and consecutively integration of *S*. *epidermidis* into the biofilm architecture. To test this hypothesis, PIA was semi-quantitatively detected in ultrasound cell wall extracts and in culture supernatants of *S*. *epidermidis* 1457 and 1457Δ*sbp* (Fig. 8A). Indeed, quantification of cell wall associated PIA in 1457Δ*sbp* by dot blot analysis found at least 32-fold reduced PIA-amounts compared to the wild type (Fig. 8A), while *in trans* expression of *sbp* restored PIA quantities to wild type levels (Fig. 8A).

If inactivation of *sbp* results in impaired recruitment of PIA to the cell surface, a major fraction should appear in culture supernatants. However, similar to the cell wall associated fractions, a clear reduction of PIA amounts was also evident in supernatants of 1457Δ*sbp* overnight cultures (Fig. 8A). Thus, 1457Δ*sbp* produced overall reduced amounts of PIA, which could be a contributing factor to the impaired biofilm forming capacity. In line with this, quantitative RT-PCR demonstrated a significant, 20-fold down regulation of *icaA* transcription in 1457Δ*sbp* compared to the wild type. *In trans* expression of *sbp* in 1457Δ*sbp* abrogated the *icaA* down regulation, and even a slight, 1.3-fold increase in expression levels was observed (S8 Fig.).

The observed down-regulation of *icaADBC* and consequently reduced PIA production in mutant 1457Δ*sbp* made it impossible to evaluate if Sbp is structurally necessary for PIA-dependent biofilm formation in *S*. *epidermidis* wild type 1457. Therefore, biofilm formation was tested in 1457-M10 and 1457-M10Δ*sbp*, each complemented with plasmid pTX*icaADBC*, allowing for inducible expression of *icaADBC* and PIA synthesis [31,32]. Induction of PIA synthesis in 1457-M10Δ*sbp*(pTX*icaADBC*) induced biofilm formation to a quantitatively identical level to that of 1457-M10(pTX*icaADBC*) (Fig. 8B). Moreover, quantification of PIA in this pair of strains retained on the cell surface and released into the supernatant found no differential distribution of PIA depending on the presence or absence of Sbp. Thus, PIA recruitment to the cell surface and subsequent biofilm accumulation appear functionally independent from Sbp, while clearly the protein has an indirect impact on PIA-dependent biofilm formation by influencing *icaADBC-*transcription and subsequent PIA synthesis.

### Interconnection of Sbp with Aap-dependent biofilm formation

**Fig 9:**
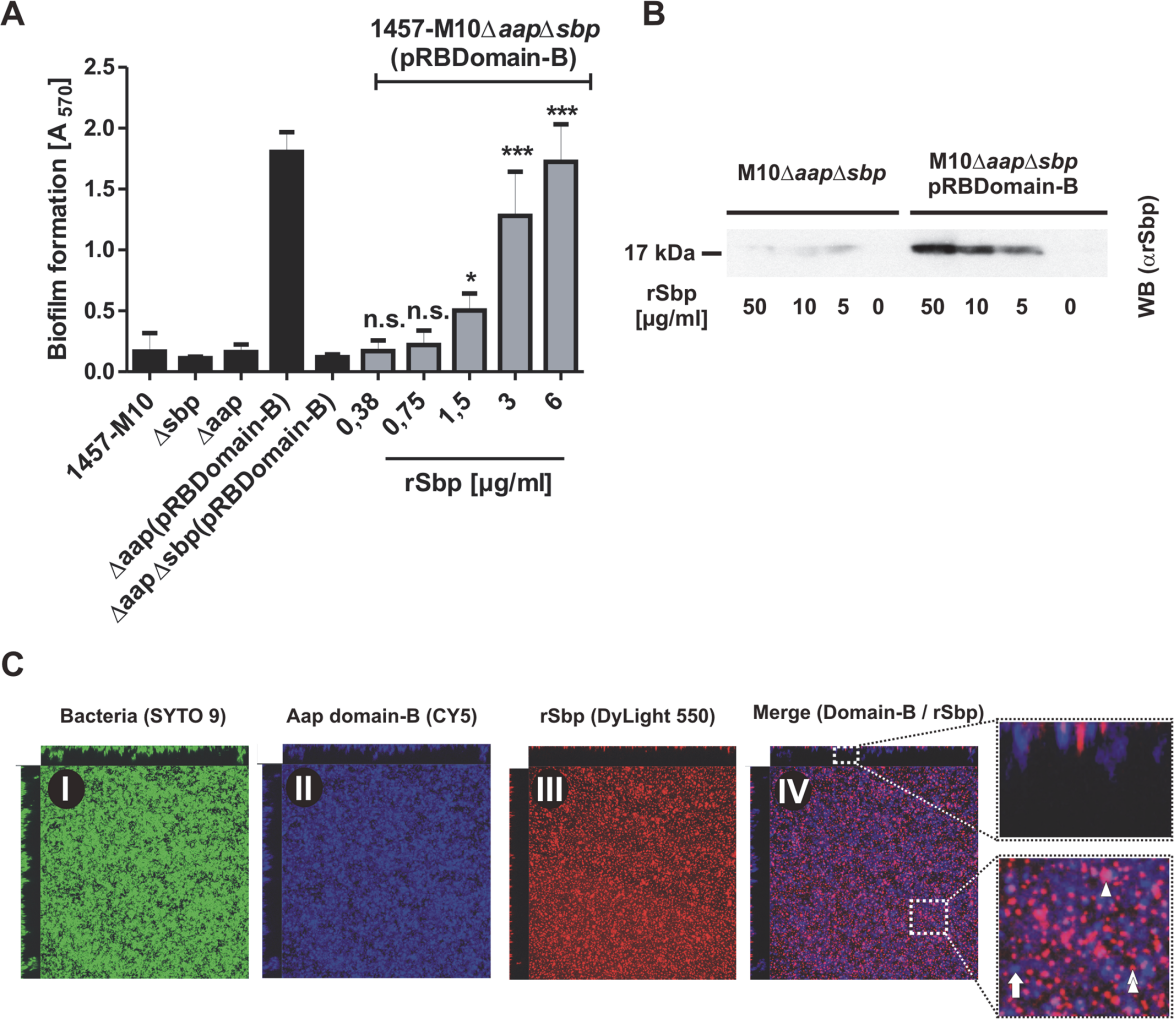
Functional role of Sbp in Aap-mediated *S*. *epidermidis* biofilm formation. **(A)** Biofilm phenotypes of 1457-M10, 1457-M10Δ*aap*, 1457-M10Δ*sbp*, 1457-M10Δ*aap*Δ*sbp*, 1457-M10Δ*aap*(pRBDomain-B) and 1457-M10Δ*aap*Δ*sbp*(pRBDomain-B) were tested in static biofilm assays in the absence (black columns) or presence of varying rSbp concentrations (grey columns). All columns represent mean of 12 values obtained in 3 independent experiments. Error bars represent standard deviations. Significant differences (p< 0.05; one-way ANOVA with Dunnett’s correction for multiple testing) are indicated (*, p<0.05; ***, p<0.001). n.s., not significant. **(B)** Recruitment of rSbp to the surface of 1457-M10Δ*aap*Δ*sbp* in the presence of absence of *in trans* expressed Aap domain-B. Western blot of cell surface protein extracts from identical numbers of bacteria suspended in PBS containing 50, 10, or 5 μg/ml rSbp. PBS without rSbp served as a negative control. rSbp was detected by bioluminescence using a polyclonal rabbit anti-rSbp antiserum and anti-rabbit IgG coupled to peroxidase. **(C)** Distribution of Aap domain-B and rSbp in living biofilms. 1457-M10Δ*aap*Δ*sbp*(pRBDomain-B) was grown in the presence of 1.5 μg/ml rSbp-DyLight550. Bacteria were stained with SYTO 9, Aap domain-B was detected using a polyclonal anti-rDomain-B antiserum and a Cy5-labelled anti-rabbit IgG antibody. Panels I–III represent images of each fluorescence channel, image IV is a merge depicting Aap domain-B and rSbp (IV). A zoom-in shows a representative area with Aap domain-B–rSbp co-localizations (purple; double arrow head). Arrow, Aap domain-B expressing cells without Sbp-recruitment (blue); arrow head; Sbp deposition independent from Aap domain-B (red).

We next asked if Sbp plays a role in Aap-dependent biofilm formation. Inactivation of *aap* in *S*. *epidermidis* 1457 and 1457Δ*sbp* had no further impact on biofilm quantity (Fig. 6A), most likely due to residual PIA synthesis in those strains (Fig. 8A), masking potential Aap effects. In line with this, *in trans* expression of *sbp* in 1457Δ*aap*Δ*sbp* not only restored quantitative PIA production (Fig. 8A)Δbut also induced biofilm formation at wild type levels (Fig. 6A). To allow for a precise analysis of Sbp function during Aap-dependent biofilm formation without confounding PIA-effects, plasmid pRBDomain-B was transduced into PIA-, Aap-, and biofilm-negative mutant 1457-M10Δ*aap*. As expected [16], *in trans* expression of Aap domain-B induced biofilm formation in 1457-M10Δ*aap*(pRBDomain-B) (Fig. 9A). In sharp contrast, *in trans* expression of Aap domain-B in *sbp* / *aap* double knock-out mutant 1457-M10Δ*aap*Δ*sbp* did not induce a biofilm-positive phenotype (Fig. 9A). Exogenous addition of rSbp, however, restored biofilm formation to levels of 1457-M10Δ*aap*(pRBDomain-B) (Fig. 9A), demonstrating that loss of biofilm forming capacity in 1457-M10Δ*aap*Δ*sbp*(pRBDomain-B) indeed was related to inactivation of *sbp*. Importantly, while 1457-M10Δ*aap*(pRBDomain-B) formed cell aggregates, the Sbp-negative 1457-M10Δ*aap*Δ*sbp*(pRBDomain-B) was unable to assemble large clusters, indicative of a defect in intercellular adhesion (Fig. 6C). Since Aap domain-B production was identical in 1457-M10Δ*aap*Δ*sbp*(pRBDomain-B) and 1457-M10Δ*aap*(pRBDomain-B) (S9 Fig.), these data indicate that Sbp is necessary for Aap-dependent cell aggregation and subsequent biofilm formation.

Following the hypothesis that Aap-dependent cell aggregation results from direct Aap domain-B interactions with Sbp on the bacterial cell surface, we tested the ability of Aap domain-B expressing 1457-M10Δ*aap*Δ*sbp*(pRBDomain-B) to recruit exogenous rSbp to the cell surface in comparison to Aap-negative 1457-M10Δ*aap*Δ*sbp*. After one hour incubation in the presence of varying amounts of rSbp, Aap-negative 1457-M10Δ*aap*Δ*sbp* did not recruit significant levels of rSbp to the cell surface (Fig. 9B). In sharp contrast, Western blotting demonstrated that *in trans* expression of Aap domain-B boosted the ability of 1457-M10Δ*aap*Δ*sbp* to bind soluble rSbp (Fig. 9B), thus demonstrating that indeed expression of Aap domain-B promotes recruitment of Sbp to the bacterial cell surface.

To assess the relevance of Aap domain-B–Sbp interactions during biofilm formation in more detail, CLSM analysis of living biofilms was performed. In order to detect Aap domain-B and Sbp in parallel, 1457-M10Δ*aap*Δ*sbp*(pRBDomain-B) was grown in the presence of 1.5 μg/ml rSbp labelled with DyLight 550, while Aap domain-B was detected by rabbit anti-rDomain-B and anti-rabbit IgG coupled to Cy5. In cultures of 1457-M10Δ*aap*Δ*sbp*(pRBDomain-B) rSbp-DyLight550 produced a film-like structure at the basal biofilm layers (Fig. 9C). Moreover, similar to the observations made with endogenously produced Sbp, there were huge aggregated Sbp clusters and spikes arising from the substrate interspersed into the biofilm architecture (Fig. 9C, panel III). Aap domain-B was detected on *S*. *epidermidis* cell surfaces throughout all levels of the biofilm (Fig. 9C, panel II). The relative spatial distribution of Sbp and Aap domain-B was analysed in merged CLSM images. Here, although by visual assessment no strict association was found, co-localization of both proteins became evident (Fig. 9C, panel IV). Indeed, bioinformatics image analysis found a high degree of Sbp-Aap co-localization (Menders coefficient M1 [Sbp-Aap domain-B]: 100%; M2 [Aap domain-B-Sbp]: 75%), lending further support to the assumption that Sbp-Aap domain-B interactions occur in living biofilms.

### Role of sbp during foreign-material infection

**Fig 10:**
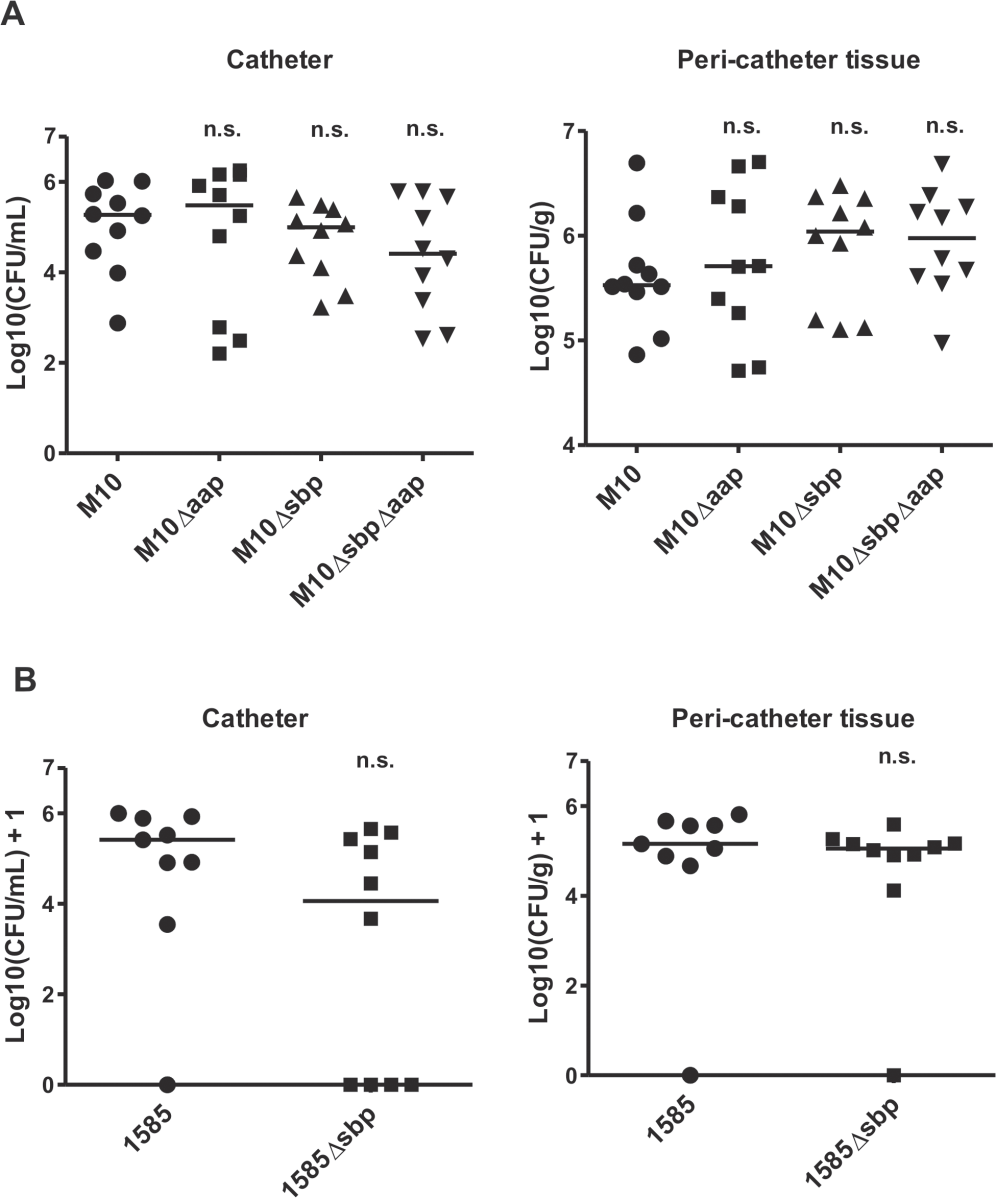
Relevance of Sbp in a mouse catheter infection-model. Implanted catheter segments were injected with **(A)** 1457-M10, 1457-M10Δ*aap*, 1457-M10Δ*sbp*, and 1457-M10Δ*aap*Δ*sbp* (n = 10 mice per strain) or **(B)** 1585 (n = 9 mice) and 1585Δ*sbp* (n = 10 mice). Animals were sacrificed after 7 days and numbers of catheter adherent cells [CFU/ml] and bacteria from the peri-catheter tissue [CFU/g] were enumerated. n.s., not significant compared to the Sbp producing parent strain (1457-M10 or 1585, respectively).

The function of Sbp during establishment of device-associated infections was tested in a mouse catheter infection model. Here, focusing on the role of Sbp in PIA-independent biofilms, the PIA-negative mutant 1457-M10 and derivatives 1457-M10Δ*aap*, 1457-M10Δ*sbp*, and 1457-M10Δ*aap*Δ*sbp* were used to exclude potential confounding effects of PIA production. After 7 days, in animals infected with 1457-M10, median recovery from catheters was 1.86 x 10^5^ CFU/ml (7.60 x 10^2^ CFU/ml—1.07 x 10^6^ CFU/ml) and 3.37 x 10^5^ CFU/g (range 7.35 x 10^4^ CFU/g—4.96 x 10^6^ CFU/g) from peri-catheter tissue. At that time point, similar CFU counts were isolated from animals infected with 1457-M10 Δ*aap* (catheter range 1.60 x 10^2^ CFU/ml—1.80 x 10^6^ CFU/ml, tissue range 5.15 x 10^4^ CFU/g—5.08 x 10^6^ CFU/g) (Fig. 10). In animals infected with 1457-M10 Δ*sbp*, median recovery from catheters was 1.00 x 10^5^ CFU/ml (range 1.68 x 10^3^ CFU/ml—4.50 x 10^5^ CFU/ml), and 1.10 x 10^6^ CFU/g (range 1.27 x 10^5^ CFU/g—3.01 x 10^6^ CFU/g) from peri-catheter tissue. Similar quantities were obtained with 1457-M10Δ*aap*Δ*sbp* (catheter range 3.40 x 10^2^–6.10 x 10^5^, tissue range 9.47 x 10^4^–4.83 x 10^6^) (Fig. 10). There was a trend towards lower numbers of catheter-associated bacteria and higher bacterial numbers in the peri-catheter tissue in 1457-M10Δ*sbp* and 1457-M10Δ*aap*Δ*sbp*, however the values did not reach statistical significance when compared to 1457-M10 (Fig. 10).

For further confirmation, the above model was also performed using *S*. *epidermidis* strain 1585, which naturally lacks both *aap* and the *ica* operon, and its isogenic mutant, 1585Δ*sbp*. Seven days after infection, 8 of 9 catheters from mice inoculated with wild-type 1585 were colonized (median recovery from catheters of 2.6 x 10^5^ CFU/ml), while bacteria were only recovered from 6 of 10 catheters from the 1585Δ*sbp* group (median recovery from catheters of 1.6 x 10^4^ CFU/ml) (Fig. 10B). Bacterial burden in the peri-catheter tissue was similar between 1585 and 1585Δ*sbp* (medians of 1.4 x 10^5^ and 1.1 x 10^5^ CFU/g tissue, respectively) (Fig. 10B). As with the 1457-M10 mutant strains, results did not reach statistical significance, however the same trend of decreased catheter-associated bacteria in the absence of Sbp was clear. In light of the *in vitro* findings, these results are not entirely surprizing, as Sbp is an important co-factor in the biofilm structure, but does not independently mediate biofilm formation.

## Discussion

**Fig 11:**
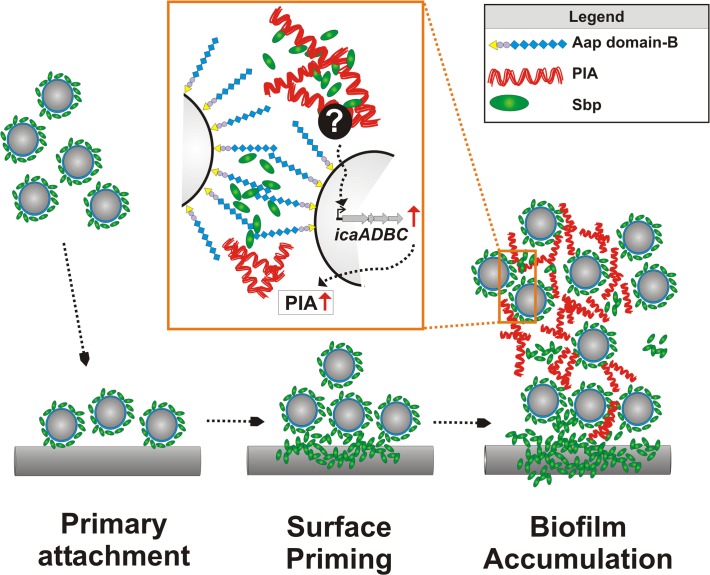
Integrated model of Sbp functions in *S*. *epidermidis* biofilm formation. Free-floating *S*. *epidermidis* decorated with cell surface bound Sbp adhere to artificial surfaces. The **fast primary attachment phase** is apparently independent from Sbp. While *S*. *epidermidis* adheres to the surface, Sbp localizes to the bacterial–substrate interface. Sbp deposition is a **surface priming process** necessary for stable *S*. *epidermidis*–foreign material interactions and sustained adherence during **biofilm accumulation**. Most likely, priming and accumulation are processes running in parallel. Sbp is part of the extracellular biofilm matrix, partly co-localizing with PIA (**Zoom in**). PIA-dependent biofilm formation indirectly depends on the presence of Sbp that, via so far unknown mechanisms modulates *icaADBC* transcription and subsequent PIA synthesis. In addition, Sbp serves as a necessary factor during Aap domain-B mediated bacterial aggregation, potentially through direct molecular interactions. Here, the involvement of additional (protein) factors cannot be excluded.

Biofilm formation is regarded as the major virulence mechanism enabling *S*. *epidermidis* to cause devastating implant-associated infections [11]. Here we describe the novel extracellular 18 kDa Sbp as a key biofilm matrix component with crucial importance for the development of a higher-order biofilm architecture. The dynamic biofilm assembly process essentially depends on bacterial mechanisms which ultimately promote cell–cell adhesion and robust cell aggregation, thereby stabilizing the multilayered biofilm consortium [13]. So far, specific constituents of the biofilm matrix that are functionally involved in biofilm accumulation, i.e. PIA, Aap, or Embp, all directly govern intercellular adhesion, and as a consequence their expression is independently inducing cell cluster formation [16,17,21] (Fig. 11). In sharp contrast, functional and spatial characterization of Sbp supports a model in which biofilm matrix-associated Sbp does not directly induce cell aggregation, but forms a biofilm scaffold that markedly fosters PIA- and Aap-mediated biofilm accumulation (Fig. 11). Thus, Sbp represents a new functional class of *S*. *epidermidis* biofilm matrix proteins, being necessary co-factors for additional intercellular adhesins. In fact, Sbp is the first structural *S*. *epidermidis* extracellular protein with significant relevance for both protein and exopolysaccharide-dependent biofilm formation.

Despite the key importance of the extracellular matrix for *S*. *epidermidis* biofilm formation, we are only beginning to understand its structural complexity and the importance of potential spatial events and a specific molecular architecture leading to its development. So far, studies investigating *S*. *epidermidis* strains which use PIA, Aap, or Embp as intercellular adhesins provided the first evidence that distinct intercellular adhesins could significantly differ in their spatial distribution [12]. While PIA was evenly distributed throughout the biofilm in horizontal and vertical dimensions, only very small amounts of proteinaceous intercellular adhesins (i.e. Aap, Embp) were found in the intercellular matrix but predominantly localized to the bacterial cell surface. In contrast, in depth analysis by CLSM images showed that Sbp is deposited within the extracellular matrix where the protein exhibits a distinct spatial distribution pattern. This finding clearly demonstrates that the *S*. *epidermidis* biofilm matrix is not a random accumulation of extracellular material but at least partially, a structured complex of polysaccharides and proteins, in which segregated matrix components potentially can take over distinct functions during biofilm development. The significant importance of a spatial- and time-resolved expression profile for bacterial biofilm formation was only recently demonstrated by showing that in *Vibrio cholerae* Vibrio polysaccharide (VPS) and biofilm matrix proteins RbmA, RbmC, and Bap1 proteins carry complementary architectural roles that ultimately shape the biofilm ultrastructure [33,34]. Notably, protein Bap1 was concentrated at the substrate of a *V*. *cholerae* biofilm, promoting bacterial adherence and tight tethering to the surface [33,34], and modification of physico-chemical surface properties by bacterial proteins has also been demonstrated for *Bacillus subtilis* protein BslA [35]. Since accumulation at the biofilm–surface interface was also found for Sbp, it is reasonable to speculate that Sbp could function as a general means used by *S*. *epidermidis* to prime surfaces during colonization. In line with this, immobilized Sbp significantly promoted stable tethering of mature biofilms on a polystyrene surface that is otherwise resistant to bacterial binding (Fig. 11). The ways used by *S*. *epidermidis* to realize the distinct spatial Sbp distribution pattern are currently unknown but could involve differential *sbp* expression by distinct *S*. *epidermidis* subpopulations and / or concerted protein assembly events. The latter possibility is favoured by the finding that distribution pattern of exogenously added rSbp and endogenous Sbp were almost indistinguishable.

Sbp was affinity-purified from crude biofilm matrix preparations using Aap domain-B as a receptor, and our additional biochemical analysis demonstrated evidence for direct Sbp–Aap domain-B interactions. So far, despite being long recognized as an intercellular adhesin, the molecular mechanisms used by Aap domain-B to induce bacterial aggregation are not well understood. Only recently the crystal structure of a sub-domain from *S*. *aureus* Aap homologue SasG was resolved, determining the potential structural basis for the stable elongated organization of Aap [27,28,30]. Analysis of recombinant Aap domain-B fragments consisting of 1, 1.5 and 2.5 G5 repeat domains by analytical ultracentrifugation and circular dichroism showed that domain-B possesses self-associating properties [29]. These homotypic G5-repeat domain interactions were dependent on the presence of zinc, suggesting a “zinc-zipper”-like mechanism for G5 domain-based *S*. *epidermidis* biofilm formation. Importantly, as reflected by specific interactions with Sbp, our results highlight that in addition to its evident self-aggregating properties, Aap domain-B also clearly possesses heterophilic binding activities. These are apparently not restricted to Sbp, since during affinity purification, major *S*. *epidermidis* autolysin AtlE was enriched in addition to Sbp. While the exact modalities of Aap domain-B–AtlE interactions and their relevance for biofilm formation remain to be elucidated, our findings indicate that Sbp is necessary for Aap domain-B mediated biofilm formation, and most likely the direct Aap domain-B–Sbp interactions play a pivotal role (Fig. 11). Indeed, this hypothesis is further strengthened by CLSM analysis, providing clear evidence for Sbp–Aap co-localizations in living biofilms. Nevertheless, at present it also has to be taken into account that Sbp, apart from being directly involved in Aap mediated cell-cell adhesion, could also exert its effects on Aap-dependent biofilm formation via indirect modalities. These could, for example, involve recruitment of additional, yet unknown factors to the bacterial cell surface which then might serve as ligands for Aap. Future studies must therefore address the question as to which extended specific multi protein complexes exist in *S*. *epidermidis* biofilms, identify the components and dissect their architecture and specific functions.

Importantly, apart from directly supporting *S*. *epidermidis* cell aggregation, Sbp clearly also has indirect effects on biofilm formation as demonstrated by the impact on PIA production. PIA is of key importance for *S*. *epidermidis* biofilm formation, and it has been speculated that it integrates bacteria into the biofilm consortium through mono- or polyvalent interactions with charged cell surface structures [36]. Indeed, there is evidence that in *E*. *coli*, PIA homologue PNAG binds to charged LPS [37]. Thus, given its cell surface localization and anticipated charged character, Sbp appeared as a potential receptor for PIA. However, in depth analysis showed that the impaired biofilm phenotype of 1457Δ*sbp* was unrelated to insufficient recruitment of PIA to the *S*. *epidermidis* cell surface, but most likely resulted from an overall reduced PIA production related to a down-regulation of *icaADBC* expression (Fig. 11). Given the stringent coupling of PIA synthesis and environmental conditions [38], the down-regulation of *icaADBC* in 1457Δ*sbp* could indicate a role of Sbp in general maintenance of biofilm matrix dependent, extracellular conditions favouring *icaADBC* expression, e.g. by ensuring a constant environmental milieu by sorption of ions or nutrients [13]. Identification of matrix derived signals stimulating *S*. *epidermidis* biofilm formation in general and the potential involvement of Sbp in this process will be of major importance in the future.

The analysis of the *in vivo* relevance of Sbp for *S*. *epidermidis* catheter colonization in a mouse catheter infection-model produced ambiguous results. Despite a slight trend towards lower catheter adherent bacterial counts in Sbp-negative mutants derived from *S*. *epidermidis* 1457-M10 and 1585, the observed differences compared to their Sbp producing parent strains did not reach statistical significance. The failure to demonstrate a clear reduction in colonization capacities of the Sbp-negative mutant is most likely related to the tremendous multitude of factors contributing to *S*. *epidermidis* biofilm formation. Apparently, the existence of partially interdependent, but also independent mechanisms that can functionally compensate for each other makes it virtually unlikely that inactivation of a single factor necessarily has a dramatic impact on virulence *in vivo*. This is in particular true for PIA-independent biofilm formation for which at least three independent biofilm mechanisms have been documented *in vitro*: Embp [17,26], Aap [16,29], and release of eDNA [18,26]. In fact, even for PIA-dependent biofilm formation, animal studies revealed conflicting results, showing that in some models *icaADBC* was necessary for virulence [9,10,25,39], while in others it was not [40,41]. Thus, for a balanced relevance assessment of distinct biofilm factors, future studies must focus on the analysis of defined mutants defective in various combinations of molecules contributing to biofilm formation, which need to be tested in different models of device-associated infections (e.g. central venous catheter- vs. orthopaedic device- infection).

In conclusion, we here show that Sbp plays a role in *S*. *epidermidis* surface colonization and biofilm formation. Thus, it is reasonable to speculate that the protein is of relevance to both extremes of *S*. *epidermidis* life styles: lifelong commensalism on human epidermal surfaces and infection after device implantation. The detailed analysis of factors playing dual roles in both of these scenarios and the well-balanced appreciation of their beneficial and potentially aggressive properties will clearly feed our general understanding of how commensal bacteria and specifically *S*. *epidermidis* turn into a “accidental” opportunistic pathogens [11].

## Material and Methods

### Bacterial strains

Strains and mutants used in this study are summarized in Table 1. For maintenance, wild type strains and markerless mutants were grown on Columbia blood agar containing sheep erythrocytes (Oxoid, Basingstoke, UK). Otherwise, bacteria were grown on TSA supplemented with chloramphenicol (10 μg/ml) or erythromycin (20 μg/ml) where necessary.

### Adherence assay and *in vitro* biofilm formation

Rapid primary attachment after 1 hour to cell culture treated polystyrene (NunclonΔ; Nunc, Roskilde, Denmark) and unmodified *n*on-*a*dhesive *p*olystyrene (referred to as NAP; Greiner, Frickenhausen, Germany) was assessed by using a specific ELISA [42]. In order to quantify *S*. *epidermidis* adherence to NAP plates (Greiner, Frickenhausen, Germany) after prolonged incubation periods (i.e. 8 and 24 hours, respectively), Gfp-expressing *S*. *epidermidis* 1457-M10(pGFP) and 1457-M10Δ*sbp*(pGFP) were grown in 150 μl TSB at 37°C under static conditions. After recording the optical density at 600 nm (A_600_) as a function of bacterial growth, the medium was discarded and non-adherent cells were removed by washing two times with 150 μl PBS. Surface adherent fluorescence intensities were immediately determined at 485 nm excitation and 535 nm emission wavelength using a spectrophotometer (Infinite M200, Tecan, Männedorf, Switzerland) in top read operating mode. Validation experiments using defined bacterial numbers that were, prior to fluorescence measurement, collected on the bottom of the micro titer plate by centrifugation revealed a linear association between fluorescence intensity and cell numbers (S10 Fig.), thus allowing to use fluorescence intensity as a function of polystyrene adherent bacteria. Calculation of adherent bacteria numbers was done by using the formula: CFU = 2562*(fluorescence intensity [AU] – 508). All fluorescence intensities were normalized against bacterial cell densities using the formula Normalized fluorescence = fluorescence [AU]/A_600_.

Biofilm formation was tested in TSB (Trypticase soy broth, Becton Dickinson, Cockeysville, USA) using the semi-quantitative microtiter plate test (biofilm assay) as described in [42], or a flow cell-based flow assay, using TSB supplemented with 1% glucose, uncoated three-channel flow cells (Stovall, USA), and a flow rate of 0.5 ml/min for 48 h. Biofilm-positive *S*. *epidermidis* 1457 [43] and its corresponding, biofilm-negative *icaADBC* transposon mutant 1457-M10 [44] served as positive and negative controls, respectively. Adhesion of *S*. *epidermidis* to keratinocytes was carried out as described in [45]. HaCaT cells were obtained from the German Cancer Research Center (Heidelberg, Germany). Confluent layers of HaCaT cells were infected with *S*. *epidermidis* at an MOI of 100. After 90 minutes of co-incubation at 37°C, cultures were washed twice with PBS to remove unbound bacteria. Keratinocytes were subsequently detached by adding 0.5% Trypsin (Biochrom), lysed by sonication at 50W for 15 seconds in H_2_O, and adherent bacteria were determined by plating out serial dilutions of HaCaT cell lysates on sheep blood agar.

### Confocal laser scanning microscopy and analysis of spatial fluorescence distribution by planimetry

Confocal acquisition was performed on a Zeiss Axiovert 200M inverted microscope equipped with a Yokogawa CSU-22 confocal head and a Hamamatsu C9100-02 EM-CCD camera. Images were taken with a Zeiss Plan Apochromat 63x/1.4 Ph3 Oil objective. Improvision Volocity software was used for image acquisition, quantification and co-localization analysis.

For quantifying the distribution of the labelled components in the biofilm, 5 random positions were recorded per condition with a z-spacing of 0.5 μm. The fluorescent structure was detected in every plane and the occupied area determined. Stacks were aligned and the obtained values plotted against the distance from the coverslip. Experiments were performed 2 times.

### Construction of *sbp* mutants by allele gene replacement and phage transduction

An allele replacement strategy was used for construction of specific *sbp* knock-out mutants in *S*. *epidermidis* 1457 and 1585 (S5A Fig.). To this end two fragments flanking regions up-stream (primers sbp_att_for 5´-GGGGACAAGTTTGTACAAAAAAGCAGGCTTATATCCTGTCGTACTCGTG-3′ and sbp_eco_rev 5′-ACCGCCGAATTCTCACTCCTTTGATTCTTTATGTCTTCTG-3′) and down-stream (primers sbp_eco_for 5′-ACCGCCGAATTCAAAGATAAAAATGTGAAGTTATATCGTA-3′ and sbp_att_rev 5′-GGGGACCACTTTGTACAAGAAAGCTGGGTAGTACGTGCAGATAAACGT-3′) of the Sbp-coding region were amplified using Phusion high fidelity polymerase (New England Biolabs, Frankfurt, Germany). Amplicons were cleaved using *Eco*RI, and the resulting, *sbp*-flanking fragments were ligated using standard procedures. Making use of 5′ and 3′ attB sites ligation products were introduced into pKOR1 [46] using the BP clonase (Invitrogen, Karlsruhe, Germany), resulting in plasmid pKO*sbp*. pKO*sbp* was by electroporation subsequently introduced first into *S*. *aureus* RN4220 and then *S*. *epidermidis* mutant 1457-M12 [32]. Next, using phage A6C, pKO*sbp* was transduced into *S*. *epidermidis* 1457 and 1585. Selection of mutants was performed essentially as described [46]. Correctness of mutations was verified by PCR, sequence analysis and Western blotting experiments demonstrated the mutant’s lost ability to produce Sbp (S5B–S5C Fig.). For complementation of the *sbp* knock-out, the anticipated natural *sbp* promoter and the *sbp* coding sequence was amplified using Phusion high fidelity polymerase and primers sbp_for_Hind (5′-CTATGTTAAGCTTttttactataagcgctgaaacagttg-3′; *Hin*dIII restriction site underlined) and sbp_rev_Eco (5′-CTATGTT*GAATTC*CTATTTTATCTTATAAAACGTATATCCA-3′; *Eco*RI restriction site underlined) and cloned into plasmid pRB473 [47], resulting in pRB*sbp*. pRB*sbp* was introduced into 1457Δ*sbp* and 1585Δ*sbp* as outlined for pKO*sbp*.

In order to generate an *aap* knock-out in an *sbp*-negative background *aap*:*tetM* was transduced from 1457Δ*aap* [48] into 1457Δ*sbp* using phage A6C, resulting in 1457Δ*aap*Δ*sbp*. In addition, *icaA*::Tn*917* was transduced from 1457-M10 into 1457Δ*aap*Δ*sbp*, giving 1457-M10Δ*aap*Δ*sbp*. 1457Δ*aap*Δ*sbp* and 1457-M10Δ*aap*Δ*sbp* were complemented with pRB*sbp*, pRB*aap*, or pRBDomain-B by phage transduction of the respective plasmids from 1585Δ*sbp*(pRB*sbp*), 1585(pRB*aap*), and 1585(pRBDomain-B) [16]. Table 1 gives a summary of mutants created in this study.

### Expression, purification and labelling of recombinant proteins

Expression and purification of recombinant Aap domain-B was performed as described [16,23]. In order to express recombinant *sbp* without its export signal, a PCR amplicon was generated using primers sbp_64 for (5’-CACCAACAACGTTGAAGCGGCAACT-3’) and sbp_510 rev (5’-TTATTTATTTAAGTCTATACGATATAACTTCACAT-’3). The amplicon was ligated into vector pENTR/D-TOPO (Invitrogen, Karlsruhe, Germany) and subcloned into expression vector pDEST17 using the clonase protocol according to the manufacturer’s recommendations. The resulting construct pDEST_*sbp*, allowing for expression of the Sbp 6 histidine residues, was introduced into *E*. *coli* BL21AI. Recombinant protein r*Sbp* was affinity purified using HiTrap chelating HP columns (GE Life Sciences, Freiburg, Germany). SDS-PAGE proved purity of the protein.

Biotinylation of rDomain-B was carried out using the ECL Biotinylation Module (GE Life Sciences, Freiburg, Germany) essentially following the manufacturer’s recommendations. In brief, 2.5 ml of rDomain-B (1 mg/ml) in bicarbonate buffer was incubated with 100 μl of biotinylation reagent. After incubation for one hour at room temperature, unbound biotin was removed by filtration through a Sephadex G25 column. Labeled protein was stored at 4°C. For fluorescence labeling of rSbp the DyLight 550 Amine-reactive dye kit (Thermo Scientific, Bonn, Germany) was used following the manufacturer’s protocol. In summary, 40 μl of borate buffer (0.67 M) was added to 0.5 ml rSbp (2 mg/ml in PBS) and the solution was transferred to a DyLight reagent vial. After one hour incubation at room temperature unbound dye was removed by loading the reaction onto a spin column. Labeled protein was stored at 4°C.

### Production of rSbp-specific rabbit antiserum

For generation of specific antisera, rabbits were, after obtaining preimmune sera, subcutaneously immunized with 100 μg of rSbp emulsified in Freund’s complete adjuvant. A booster immunization was performed after 4 weeks using 100 μg of rSbp emulsified in Freund’s incomplete adjuvant. After an additional 4 weeks the rabbit was sacrificed and serum was stored at −20°C. Western blotting using rSbp used for immunization proved the lack of Sbp-specific antibodies in the preimmune sera, whereas the sera obtained after immunization strongly reacted with the rSbp.

### Preparation of *S*. *epidermidis* extracellular proteins, Western blot and dot blot analysis

For preparation of cell wall associated, non-covalently linked proteins, bacterial cells were grown over-night in NunclonΔ dishes (9 cm diameter), washed in PBS and, if applicable, adjusted to identical cell densities by absorbance measurement at 600 nm (A_600_). Bacterial cells were harvested from identical volumes, resuspended in LDS buffer (Invitrogen, Karlsruhe, Germany) and boiled for 5 minutes. After centrifugation, the supernatant was recovered for further analysis. Alternatively, for preparation of cell surface associated proteins, bacteria were grown over-night in 10 ml TSB in cell culture dishes (9 cm diameter, Nunc, Roskilde, Denmark). Bacterial cells were scrapped off the surface, washed once with PBS and were finally re-suspended in 5 ml PBS. Proteins were then removed from the bacterial surface by sonification (30 sec., Branson Ultrasonifier, Danbury, USA). Sonification was validated for not lysing cells by testing for viable cell numbers before and after ultrasound exposure, ensuring that no significant amounts of intracellular proteins were released. Ultra-sound extracts were cleared by centrifugation and the supernatants were further analyzed. Protein concentrations were determined by using the Lowry-assay according to the manufacturer’s instructions (Bio-Rad, Munich, Germany). Western blotting was performed by transfer of proteins separated by SDS-PAGE onto PVDF membranes. After blocking of membranes (Protein free blocking agent, Pierce, Rockford, USA), Sbp was detected by a polyclonal rabbit anti-rSbp antiserum diluted 1:20,000 in PBS buffer containing 0.01% [vol/vol] Tween 20. Bound anti-rSbp IgG were then detected by chemiluminescence (ECL, GE Life Sciences, Freiburg, Germany) after incubation with a goat anti-rabbit IgG coupled to peroxidase. SDS- polyacrylamide gel electrophoresis (SDS-PAGE), and mass spectrometry have been described elsewhere [49]. PIA production was performed essentially as described [42]. PIA was detected by chemiluminescence using wheat germ agglutinin coupled to peroxidase (Sigma Aldrich, Munich, Germany).

### Affinity purification of rDomain-B interaction partners, recruitment of rSbp to the cell wall, analysis of protein—protein interactions

1 ml freshly expressed and purified rDomain-B (10 mg/ml) in coupling buffer (0.2 M NaHCO_3_, 0.5 M NaCl, pH 8.3) was loaded onto a 1 ml HiTrap NHS-activated HP column (GE Life Sciences, Freiburg, Germany). After 30 minutes of incubation at room temperature the column was alternating rinsed with 3 x 2 ml buffer A (0,5 M ethanolamine, 0,5 M NaCl, pH 8,3) or B (0,1 M acetate, 0,5 M NaCl, pH 4). After three cycles of washing the column was finally equilibrated with 0.05 M NaHPO_4_, pH 7.4, 1 ml of crude biofilm matrix preparations suspended in PBS was loaded onto the functionalized column and incubated at room temperature for 30 minutes. After that, the column was washed with 10 ml buffer A at a flow rate of 0.5 ml/min. After that, elution buffer (Tris-HCl, pH 7.4) was applied at a flow rate of 0.5 ml/min. During all washing procedures 1 ml fractions were collected and stored at 4°C. Proteins from the collected fractions were precipitated using TCA, suspended in PBS and analyzed by SDS-PAGE.

In order to demonstrate recruitment of Sbp to the bacterial cell surface, cells of *S*. *epidermidis* strains 1457-M10Δ*aap*Δ*sbp* and 1457-M10Δ*aap*Δ*sbp*(pRBDomain-B) from an overnight culture were collected by centrifugation and washed once with PBS. Pellets were resuspended in 200 μl PBS containing different amounts of rSbp and suspensions were incubated for 1 hour at room temperature with agitation. After centrifugation, bacteria were washed twice with PBS, and pellets were finally resuspended in 1X LDS buffer (Invitrogene, Karlsruhe, Germany). After heating (5 minutes 90°C) protein extracts were analyzed by SDS PAGE and western blotting.

For analysis of rDomain-B—rSbp interactions 100 μl of rDomain-B in PBS (5 μg/ml) were loaded into the wells of a 96 well microtiter plate (Greiner, Frickenhausen, Germany). After overnight incubation at 4°C plates were washed with PBS and blocked for one hour using protein free blocking buffer (Thermo Scientific, Bonn, Germany). Next, 100 μl PBS containing rSbp at various concentrations were applied and incubated for one hour at room temperature. After washing with PBS containing Tween-20 (0.05% [vol/vol]) bound rSbp was detected using the rabbit anti-rSbp antiserum diluted 1:10,000 in PBS + Tween-20 (0.05% [vol/vol] and alkaline phosphatase- (AP-) coupled goat anti-rabbit IgG (Dianova, Hamburg, Germany) as a second antibody. After one hour incubation with AP substrate buffer (0.1 M glycine, 1 mM ZnCl_2_, 1mM MgCl_2_) containing AP substrate 477 (Sigma Aldrich, Munich, Germany) the absorption at 405 nm (reference wave length 492 nm) was estimated using an ELISA plate reader. The uncoated polystyrene surface served as a negative control. In competition experiments, PBS containing rSbp (150 μg/ml) was incubated with various amounts of rDomain-B (0.4–100 μg/ml) for one hour at 4°C. 100 μl of the mixture were loaded into rDomain-B-coated wells and binding of rSbp was estimated as described above. rSbp without prior incubation with rDomain-B served as a reference control. Inhibition of rSbp binding was calculated using the formula (1—A_405_ rSbp w/rDomain-B / A_405_ rSbp w/o rDomain-B) x 100.

### RNA preparation and transcription analysis

For RNA preparation, overnight cultures in TSB were diluted 1:100 in fresh medium and incubated in NunclonΔ 9 cm tissue culture dishes (Nunc, Roskilde, Denmark) at 37°C under static conditions [50]. Total RNA was isolated from two dishes (20 ml) as described [51]. Quality of isolated RNA was verified by an average optical density (OD) OD_260_/OD_280_ nm absorption ratio of 1,92 (range 1,75–2,01).

For transcription analysis, RNA was digested with RNase-free RQ1 DNase (Promega, Madison, USA) at a concentration of 1 U/μg RNA for 45 minutes at 37°C, and 500 ng RNA were reverse transcribed with iScript cDNA Synthesis Kit (BioRad, Munich, Germany) following the manufacturer’s instructions. PCR was performed on an iCycler thermal cycler (BioRad, Munich, Germany) in 25 μl reaction volumes using iQ SYBR Green Supermix (BioRad, Munich, Germany) and commercially generated (MWG, Munich, Germany) *icaA*—specific primers [52] at a final concentration of 300 nM each. Cycling conditions for all experiments were as follows: denaturation (95°C 5 min); 40 cycles of amplification and quantification (30 s at 95°C, 30 s at 55°C, 30 s at 72°C, plate read); melting curve (65–95°C). Gradient PCR confirmed 55°C as appropriate annealing temperature for all primers. All samples were run in triplicates in each of three independent experiments. Relative expression levels were estimated as described [53] with *gyrB* as internal control [54] and *S*. *epidermidis* 1457 as the calibrator. Identical amplification efficiency (E) for all primer pairs was assured by analyzing 10-fold serial dilutions of genomic DNA [55].

### Mouse foreign material infection model

A modification of the previously described mouse foreign material infection model [25,56] was performed under a University of Nebraska Medical Center approved Institutional Animal Care and Use Committee (IACUC) Protocol to PDF. Briefly, flanks of 8 week-old male C57BL/6 mice (National Cancer Institute) were shaved and the skin cleansed with povidone-iodine solution. A small incision was made in the skin and a one centimetre segment of polyethylene catheter was aseptically implanted subcutaneously. The incision was sealed with Vetbond (3M; Minneapolis, MN) and 10^7^ CFU (in a 20 μl volume) of *S*. *epidermidis* was injected through the skin into the catheter lumen. Seven days after inoculation, mice were sacrificed by CO_2_ inhalation, and the catheter and surrounding tissue were aseptically removed, processed, and plated to determine bacterial burden.

### Ethics statement

The immunization of rabbits using recombinantly expressed and purified rSbp was reviewed and approved by the Animal Welfare Officer of the UK Hamburg-Eppendorf, the local ethics committee and the licensing authority “Behörde für Gesundheit und Soziales (BGS) / Hamburg” (project licence number A10a/444_Immunisierung). Animals were treated in adherence to the German animal welfare law and according to international recommendations (Schweizer Bundesamt für Veterinärwesen, Richtlinie Tierschutz 3.04, 1999; Tierärztliche Vereinigung für Tierschutz: Tierschutzaspekte bei der Immunisierung von Versuchstieren, 1991; Canadian Council on Animal Care: guidelines on antibody production, 2002). The University of Nebraska Medical Center is accredited by the Association for Assessment and Accreditation of Laboratory Animal Care International (AALAC). All animals at the University of Nebraska Medical Center are maintained in accordance with the Animal Welfare Act and the DHHS “Guide for the Care and Use of Laboratory Animals.”

## Supporting Information

S1 Fig Analysis of Aap domain-A binding proteins in crude biofilm matrix preparations from *S*. *epidermidis* 1457. Matrix preparations were loaded onto a sepharose column to which recombinant Aap domain-A was coupled. Lane 1 shows the input protein preparation that was loaded onto the column. Lanes 2–8: fractions collected during washing with PBS (pH 7.4). Lanes 9–12: fractions collected during elution with Tris-HCl. Proteins were made visible by silver staining (Pierce silver stain kit). No Aap domain-A binding proteins were detected.
(TIF)

S2 Fig SDS-PAGE analysis of protein preparations from mutants 1457Δ*sarA* and 1457Δ*rna*III. Bacteria were harvested at defined time points, cell numbers were adjusted to an identical A_600_, and cells from identical volumes were harvested and resuspended in 1x LDS buffer. Proteins were extracted by incubation at 70°C for 5 minutes. Supernatants were loaded onto a 10% SDS-PAGE and proteins were stained by Coomassie.
(TIF)

S3 Fig Analysis of Sbp surface accumulation. **(A)**. Biofilm negative *S*. *epidermidis* mutant 1457-M10 was grown under static conditions in wells of a Non-adhesive polystyrol (NAP) plate (Greiner, Frickenhausen, Germany) at 37°C. After 8 and 20 hours bacterial cell densities were determined at 600 nm, and loosely adherent bacteria were then removed by washing with 200 μl PBS for four times. Remaining bacteria were then lysed by addition of 150 μl PBS containing lysostaphin (15 U/μl) and incubation at 37°C for 2 hours. After an additional PBS washing step, surfaces were blocked for 12 h at 4°C using Protein-free blocking buffer. Surface located Sbp was next detected by using a rabbit anti-rSbp antiserum and anti-rabbit IgG coupled to alkaline phosphatase, and absorption at 405 nm was measured. To normalize for differences in bacterial growth, A_405_ values were normalized against the A_600_ to give the relative Sbp amount. Sbp-negative mutant 1457-M10Δ*sbp* was used as a negative control. Relative Sbp amounts significantly differed between 8 h and 20 h (p<0.05; Bonferroni’s one-way ANOVA; ***, p<0.001). Bars show mean of nine values obtained in three independent experiments. Error bars represent standard deviation. **(B)** Spatial Sbp organization in biofilm-negative *S*. *epidermidis* 1457-M10(pGFP). Gfp-producing bacteria were grown in TSB at 37°C. After 8 h (Panel 1) and 20 h (panel 2) non-adherent bacteria were washed off and endogenous Sbp was detected using an anti-rSbp antiserum and Alexa-568 labelled anti-rabbit IgG. A continuous Sbp layer at the bacteria—substratum interface is already visible after 8 hours of growth (upper zoom-in). After 20 h of growth, additional Sbp clusters become apparent (upper zoom-in). Merged images of green (bacteria) and red (Sbp) channel are shown.
(TIF)

S4 Fig Adhesion of *S*. *epidermidis* strain 1585, 185Δ*sbp* and the complemented mutant to HaCaT keratinocytes. Columns represent mean of 10 values obtained in 5 independent experiments, error bars depict standard error of mean. Differences between 1585 and 1585Δ*sbp* as well as 1585Δ*sbp* and the complemented mutant were significant different (p<0.01, Wilcoxon rank sum test).
(TIF)

S5 Fig Construction and validation of mutant 1457Δ*sbp* and 1585Δ*sbp*. **(A)** Strategy for construction of a *sbp* gene knock-out mutant. For construction of *sbp* knock-out mutants DNA fragments located up-stream and down-stream of sbp were amplified, ligated and cloned into pKOR1. The plasmid was then introduced into S. epidermidis 1457 and used to create markerless sbp knock-out mutant 1457Δ*sbp*. **(B)** PCR analysis using primers binding up- and downstream of *sbp* (ABC_for 5′-GAAGTAGATTATACGATTACTACCATAGA-3′; CHP_rev 5′-ACTCAGAAAGATATGTCTAGCATAT-3′). A clear band shift is evident in *sbp* k.o. mutant 1457Δ*sbp*. **(C)** Western blot analysis of cell surface associated proteins from *S*. *epidermidis* wild types 1457 and 1585, the respective corresponding *sbp* knock-out mutants 1457Δ*sbp* and 1585Δ*sbp*, and the complemented mutants carrying pRB*sbp*. Sbp was detected using an anti-rSbp-antiserum and anti-rabbit IgG coupled to peroxidase.
(TIF)

S6 Fig Effect of labelled rSbp-DyLight550 on biofilm formation of mutant 1457Δ*sbp*. **(A)** Spatial analysis of rSbp-DyLight550 added to Gfp-expressing *S*. *epidermidis* 1457Δ*sbp* cultures (final concentration 1.5 μg/ml). CLSM pictures were taken after 24 hours of growth. The right panel shows a zoom into the boxed region, demonstrating the enrichment of rSbp-DyLight550 at the biofilm substratum interface as well as its presence within the biofilm matrix. Scale bars = 6 μm. **(B)** Induction of biofilm formation in 1457Δ*sbp* by labelled recombinant rSbp-DyLight 550. Similar to results obtained in experiments using unlabelled rSbp, the addition of labelled rSbp induced biofilm formation in 1457Δ*sbp* to a quantity similar to wild type 1457.
(TIF)

S7 Fig Effect of *in trans sbp* expression on biofilm phenotype of *S*. *carnosus* TM300. Constitutive expression of Sbp in TM300(pRB*sbp*) (lower panel; Sbp detected by dot immune assays [DIA] analysis of cell surface proteins using anti-rSbp) did not induce biofilm formation of biofilm-negative TM300 wt.
(TIF)

S8 Fig Relative *icaA* transcription in *S*. *epidermidis* 1457Δ*sbp* and 1457Δ*sbp*(pRB*sbp*). *icaA* transcription was assessed in three independent RNA-preparations from exponential growing cultures of 1457Δ*sbp* and 1457Δ*sbp*(pRB*sbp*) by RT-PCR. Relative *icaA* transcription was determined using wild type *S*. *epidermidis* 1457 as a calibrator. Horizontal lines indicate mean down-regulation, error bars represent SEM. Significant differences compared to *S*. *epidermidis* 1457 (One-way ANOVA with Dunnett’s correction for multiple testing) are indicated (**, p<0.01). n.s., not significant.
(TIF)

S9 Fig Detection of Aap in *S*. *epidermidis* 1457-M10Δ*aap*, 1457-M10Δ*aap*(pRBDomain-B), and 1457-M10Δ*aap*Δ*sbp*(pRBDomain-B). Cell surface proteins were separated by SDS-PAGE, blotted onto a PVDF-membrane. Aap domain-B was detectect by chemiluminescence using a rabbit anti-rDomain-B antiserum and anti-rabbit IgG coupled to POD. No apparent quantitative differences in Aap domain-B amounts between 1457-M10Δ*aap*(pRBDomain-B), and 1457-M10Δ*aap*Δ*sbp*(pRBDomain-B) are visible.
(TIF)

S10 Fig Linear association between surface localized *S*. *epidermidis* cell numbers and fluorescence intensity. In order to set up an fluorescence based assay for quantification of surface adherent bacteria 1457-M10(pGFP) was grown for 8 hours in TSB at 37°C, 180 rpm. Starting with 108 CFU suspended in 100 μl PBS, serial dilutions were prepared and 100 μl of each dilution was added to a well of a 96 well micro titer plate (Greiner, Frickenhausen, Germany). Bacteria were collected at the bottom of the plate by centrifugation, and PBS was carefully discarded. Surface adherent fluorescence was then determined at 485 nm excitation and 535 nm emission wavelength using a spectrophotometer (Infinite M200, Tecan, Männedorf, Switzerland) in top read operating mode. Fluorescence intensities [AU] were plotted against CFU, revealing a linear association described by the formula CFU = 2562x[logFU-508].
(TIF)

## References

[1] Foster CB, Sabella C. Health care—associated infections in children. JAMA 2011 Apr 13;305(14):1480–1. 10.1001/jama.2011.449 21486980

[2] Wisplinghoff H, Seifert H, Tallent SM, Bischoff T, Wenzel RP, Edmond MB. Nosocomial bloodstream infections in pediatric patients in United States hospitals: epidemiology, clinical features and susceptibilities. Pediatr Infect Dis J 2003 Aug;22(8):686–91. 12913767 10.1097/01.inf.0000078159.53132.40

[3] Wisplinghoff H, Seifert H, Wenzel RP, Edmond MB. Current trends in the epidemiology of nosocomial bloodstream infections in patients with hematological malignancies and solid neoplasms in hospitals in the United States. Clin Infect Dis 2003 May 1;36(9):1103–10. 12715303 10.1086/374339

[4] Ammerlaan HS, Harbarth S, Buiting AG, Crook DW, Fitzpatrick F, Hanberger H, et al Secular trends in nosocomial bloodstream infections: antibiotic-resistant bacteria increase the total burden of infection. Clin Infect Dis 2013 Mar;56(6):798–805. 10.1093/cid/cis1006 23223600

[5] Darouiche RO. Treatment of infections associated with surgical implants. N Engl J Med 2004 Apr 1;350(14):1422–9. 15070792 10.1056/NEJMra035415

[6] Maki DG, Kluger DM, Crnich CJ. The risk of bloodstream infection in adults with different intravascular devices: a systematic review of 200 published prospective studies. Mayo Clin Proc 2006 Sep;81(9):1159–71. 16970212 10.4065/81.9.1159

[7] Mack D, Horstkotte MA, Rohde H, Knobloch JKM. Coagulase-Negative *Staphylococci* In: Pace JL, Rupp ME, Finch RG, editors. Biofilms, Infection, and Antimicrobial Therapy.Boca Raton: CRC Press; 2006 p. 109–53.

[8] Zimmerli W, Trampuz A, Ochsner PE. Prosthetic-joint infections. N Engl J Med 2004 Oct 14;351(16):1645–54. 15483283 10.1056/NEJMra040181

[9] Rupp ME, Ulphani JS, Fey PD, Mack D. Characterization of *Staphylococcus epidermidis* polysaccharide intercellular adhesin/hemagglutinin in the pathogenesis of intravascular catheter-associated infection in a rat model. Infect Immun 1999 May;67(5):2656–9. 10225938 10.1128/iai.67.5.2656-2659.1999 PMC116021

[10] Vuong C, Kocianova S, Voyich JM, Yao Y, Fischer ER, DeLeo FR, et al A crucial role for exopolysaccharide modification in bacterial biofilm formation, immune evasion, and virulence. J Biol Chem 2004 Dec 24;279(52):54881–6. 15501828 10.1074/jbc.M411374200

[11] Otto M. *Staphylococcus epidermidis*—the 'accidental' pathogen. Nat Rev Microbiol 2009 Aug;7(8):555–67. 10.1038/nrmicro2182 19609257 PMC2807625

[12] Schommer NN, Christner M, Hentschke M, Ruckdeschel K, Aepfelbacher M, Rohde H. *Staphylococcus epidermidis* uses distinct mechanisms of biofilm formation to interfere with phagocytosis and activation of mouse macrophage-like cells 774A.1. Infect Immun 2011 Mar 14. 10.1128/IAI.01142-10 PMC3125858 21402760

[13] Flemming HC, Wingender J. The biofilm matrix. Nat Rev Microbiol 2010 Sep;8(9):623–33. 10.1038/nrmicro2415 20676145

[14] Costerton JW, Stewart PS, Greenberg EP. Bacterial biofilms: a common cause of persistent infections. Science 1999 May 21;284(5418):1318–22. 10334980 10.1126/science.284.5418.1318

[15] Mack D, Fischer W, Krokotsch A, Leopold K, Hartmann R, Egge H, et al The intercellular adhesin involved in biofilm accumulation of *Staphylococcus epidermidis* is a linear beta-1,6-linked glucosaminoglycan: purification and structural analysis. J Bacteriol 1996 Jan;178(1):175–83. 8550413 10.1128/jb.178.1.175-183.1996 PMC177636

[16] Rohde H, Burdelski C, Bartscht K, Hussain M, Buck F, Horstkotte MA, et al Induction of *Staphylococcus epidermidis* biofilm formation via proteolytic processing of the accumulation-associated protein by staphylococcal and host proteases. Mol Microbiol 2005 Mar;55(6):1883–95. 15752207 10.1111/j.1365-2958.2005.04515.x

[17] Christner M, Franke GC, Schommer NN, Wendt U, Wegert K, Pehle P, et al The giant extracellular matrix-binding protein of *Staphylococcus epidermidis* mediates biofilm accumulation and attachment to fibronectin. Mol Microbiol 2010 Jan;75(1):187–207. 10.1111/j.1365-2958.2009.06981.x 19943904

[18] Qin Z, Ou Y, Yang L, Zhu Y, Tolker-Nielsen T, Molin S, et al Role of autolysin-mediated DNA release in biofilm formation of *Staphylococcus epidermidis* . Microbiology 2007 Jul;153(Pt 7):2083–92. 17600053 10.1099/mic.0.2007/006031-0

[19] Sadovskaya I, Vinogradov E, Flahaut S, Kogan G, Jabbouri S. Extracellular carbohydrate-containing polymers of a model biofilm-producing strain, *Staphylococcus epidermidis* RP62A. Infect Immun 2005 May;73(5):3007–17. 15845508 10.1128/IAI.73.5.3007-3017.2005 PMC1087347

[20] Mack D, Davies AP, Harris LG, Knobloch JK, Rohde H. *Staphylococcus epidermidis* biofilms: functional molecules, relation to virulence, and vaccine potential. Topics in current chemistry 2009. 10.1007/128_2008_19 22328030

[21] Heilmann C, Schweitzer O, Gerke C, Vanittanakom N, Mack D, Götz F. Molecular basis of intercellular adhesion in the biofilm-forming *Staphylococcus epidermidis* . Mol Microbiol 1996 Jun;20(5):1083–91. 8809760 10.1111/j.1365-2958.1996.tb02548.x

[22] Rohde H, Kalitzky M, Kroger N, Scherpe S, Horstkotte MA, Knobloch JK, et al Detection of virulence-associated genes not useful for discriminating between invasive and commensal *Staphylococcus epidermidis* strains from a bone marrow transplant unit. J Clin Microbiol 2004 Dec;42(12):5614–9. 15583290 10.1128/JCM.42.12.5614-5619.2004 PMC535265

[23] Rohde H, Burandt EC, Siemssen N, Frommelt L, Burdelski C, Wurster S, et al Polysaccharide intercellular adhesin or protein factors in biofilm accumulation of *Staphylococcus epidermidis* and *Staphylococcus aureus* isolated from prosthetic hip and knee joint infections. Biomaterials 2007 Mar;28(9):1711–20. 17187854 10.1016/j.biomaterials.2006.11.046

[24] Ziebuhr W, Heilmann C, Götz F, Meyer P, Wilms K, Straube E, et al Detection of the intercellular adhesion gene cluster (*ica*) and phase variation in *Staphylococcus epidermidis* blood culture strains and mucosal isolates. Infect Immun 1997 Mar;65(3):890–6. 9038293 10.1128/iai.65.3.890-896.1997 PMC175065

[25] Rupp ME, Ulphani JS, Fey PD, Bartscht K, Mack D. Characterization of the importance of polysaccharide intercellular adhesin/hemagglutinin of *Staphylococcus epidermidis* in the pathogenesis of biomaterial-based infection in a mouse foreign body infection model. Infect Immun 1999 May;67(5):2627–32. 10225932 10.1128/iai.67.5.2627-2632.1999 PMC116015

[26] Christner M, Heinze C, Busch M, Franke G, Hentschke M, Bayard DS, et al *sarA* negatively regulates *Staphylococcus epidermidis* biofilm formation by modulating expression of 1 MDa extracellular matrix binding protein and autolysis-dependent release of eDNA. Mol Microbiol 2012 Oct;86(2):394–410. 10.1111/j.1365-2958.2012.08203.x 22957858

[27] Banner MA, Cunniffe JG, Macintosh RL, Foster TJ, Rohde H, Mack D, et al Localized tufts of fibrils on *Staphylococcus epidermidis* NCTC 11047 are comprised of the accumulation-associated protein. J Bacteriol 2007 Apr;189(7):2793–804. 17277069 10.1128/JB.00952-06 PMC1855787

[28] Gruszka DT, Wojdyla JA, Bingham RJ, Turkenburg JP, Manfield IW, Steward A, et al Staphylococcal biofilm-forming protein has a contiguous rod-like structure. Proc Natl Acad Sci U S A 2012 Apr 24;109(17):E1011–E1018. 10.1073/pnas.1119456109 22493247 PMC3340054

[29] Conrady DG, Brescia CC, Horii K, Weiss AA, Hassett DJ, Herr AB. A zinc-dependent adhesion module is responsible for intercellular adhesion in staphylococcal biofilms. Proc Natl Acad Sci U S A 2008 Dec 9;105(49):19456–61. 10.1073/pnas.0807717105 19047636 PMC2592360

[30] Conrady DG, Wilson JJ, Herr AB. Structural basis for Zn2+-dependent intercellular adhesion in staphylococcal biofilms. Proc Natl Acad Sci U S A 2013 Jan 15;110(3):E202–E211. 10.1073/pnas.1208134110 23277549 PMC3549106

[31] Gerke C, Kraft A, Süssmuth R, Schweitzer O, Götz F. Characterization of the N-acetylglucosaminyltransferase activity involved in the biosynthesis of the *Staphylococcus epidermidis* polysaccharide intercellular adhesin. J Biol Chem 1998 Jul 17;273(29):18586–93. 9660830 10.1074/jbc.273.29.18586

[32] Mack D, Rohde H, Dobinsky S, Riedewald J, Nedelmann M, Knobloch JKM, et al Identification of three essential regulatory gene loci governing expression of the *Staphylococcus epidermidis* polysaccharide intercellular adhesin and biofilm formation. Infect Immun 2000;68(7):3799–807. 10858187 10.1128/iai.68.7.3799-3807.2000 PMC101651

[33] Berk V, Fong JC, Dempsey GT, Develioglu ON, Zhuang X, Liphardt J, et al Molecular architecture and assembly principles of *Vibrio cholerae* biofilms. Science 2012 Jul 13;337(6091):236–9. 10.1126/science.1222981 22798614 PMC3513368

[34] Absalon C, Van DK, Watnick PI. A communal bacterial adhesin anchors biofilm and bystander cells to surfaces. PLoS Pathog 2011 Aug;7(8):e1002210 10.1371/journal.ppat.1002210 21901100 PMC3161981

[35] Hobley L, Ostrowski A, Rao FV, Bromley KM, Porter M, Prescott AR, et al BslA is a self-assembling bacterial hydrophobin that coats the *Bacillus subtilis* biofilm. Proc Natl Acad Sci U S A 2013 Aug 13;110(33):13600–5. 10.1073/pnas.1306390110 23904481 PMC3746881

[36] Vergara-Irigaray M, Maira-Litran T, Merino N, Pier GB, Penades JR, Lasa I. Wall teichoic acids are dispensable for anchoring the PNAG exopolysaccharide to the *Staphylococcus aureus* cell surface. Microbiology 2008 Mar;154(Pt 3):865–77. 10.1099/mic.0.2007/013292-0 18310032 PMC2292800

[37] Amini S, Goodarzi H, Tavazoie S. Genetic dissection of an exogenously induced biofilm in laboratory and clinical isolates of *E*. *coli* . PLoS Pathog 2009 May;5(5):e1000432 10.1371/journal.ppat.1000432 19436718 PMC2675270

[38] Mack D, Becker P, Chatterjee I, Knobloch JKM, Peters G, Rohde H, et al Mechanisms of biofilm formation in *Staphylococcus epidermidis* and *Staphylococcus aureus*: functional molecules, regulatory circuits, and adaptive responses. International Journal of Medical Microbiology 2004;294:203–12. 15493831 10.1016/j.ijmm.2004.06.015

[39] Vuong C, Voyich JM, Fischer ER, Braughton KR, Whitney AR, DeLeo FR, et al Polysaccharide intercellular adhesin (PIA) protects *Staphylococcus epidermidis* against major components of the human innate immune system. Cell Microbiol 2004 Mar;6(3):269–75. 14764110 10.1046/j.1462-5822.2004.00367.x

[40] Chokr A, Leterme D, Watier D, Jabbouri S. Neither the presence of *ica* locus, nor in vitro-biofilm formation ability is a crucial parameter for some *Staphylococcus epidermidis* strains to maintain an infection in a guinea pig tissue cage model. Microb Pathog 2007 Feb;42(2–3):94–7. 17084581 10.1016/j.micpath.2006.09.001

[41] Francois P, Tu Quoc PH, Bisognano C, Kelley WL, Lew DP, Schrenzel J, et al Lack of biofilm contribution to bacterial colonisation in an experimental model of foreign body infection by *Staphylococcus aureus* and *Staphylococcus epidermidis* . FEMS Immunol Med Microbiol 2003 Mar 20;35(2):135–40. 12628549 10.1016/S0928-8244(02)00463-7

[42] Mack D, Bartscht K, Fischer C, Rohde H, de Grahl C, Dobinsky S, et al Genetic and biochemical analysis of *Staphylococcus epidermidis* biofilm accumulation. Meth Enzymol 2001;336:215–39. 11398401 10.1016/s0076-6879(01)36592-8

[43] Mack D, Siemssen N, Laufs R. Parallel induction by glucose of adherence and a polysaccharide antigen specific for plastic-adherent *Staphylococcus epidermidis*: evidence for functional relation to intercellular adhesion. Infect Immun 1992 May;60(5):2048–57. 1314224 10.1128/iai.60.5.2048-2057.1992 PMC257114

[44] Mack D, Nedelmann M, Krokotsch A, Schwarzkopf A, Heesemann J, Laufs R. Characterization of transposon mutants of biofilm-producing *Staphylococcus epidermidis* impaired in the accumulative phase of biofilm production: genetic identification of a hexosamine-containing polysaccharide intercellular adhesin. Infect Immun 1994 Aug;62(8):3244–53. 8039894 10.1128/iai.62.8.3244-3253.1994 PMC302952

[45] Bur S, Preissner KT, Herrmann M, Bischoff M. The *Staphylococcus aureus* extracellular adherence protein promotes bacterial internalization by keratinocytes independent of fibronectin-binding proteins. J Invest Dermatol 2013 Aug;133(8):2004–12. 10.1038/jid.2013.87 23446985

[46] Bae T, Schneewind O. Allelic replacement in *Staphylococcus aureus* with inducible counter-selection. Plasmid 2006 Jan;55(1):58–63. 16051359 10.1016/j.plasmid.2005.05.005

[47] Brückner R. A series of shuttle vectors for *Bacillus subtilis* and *Escherichia coli* . Gene 1992 Dec 1;122(1):187–92. 1452028 10.1016/0378-1119(92)90048-t

[48] Schaeffer CR, Woods KM, Longo GM, Kiedrowski MR, Paharik AE, Buttner H, et al Accumulation-Associated Protein Enhances *Staphylococcus epidermidis* Biofilm Formation under Dynamic Conditions and Is Required for Infection in a Rat Catheter Model. Infect Immun 2015 Jan;83(1):214–26. 10.1128/IAI.02177-14 25332125 PMC4288872

[49] Stürenburg E, Sobottka I, Mack D, Laufs R. Cloning and sequencing of *Enterobacter aerogenes* OmpC-type osmoporin linked to carbapenem resistance. Int J Med Microbiol 2002 Mar;291(8):649–54. 12008919 10.1078/1438-4221-00175

[50] Dobinsky S, Kiel K, Rohde H, Bartscht K, Knobloch JK, Horstkotte MA, et al Glucose-related dissociation between *icaADBC* transcription and biofilm expression by *Staphylococcus epidermidis*: evidence for an additional factor required for polysaccharide intercellular adhesin synthesis. J Bacteriol 2003 May;185(9):2879–86. 12700267 10.1128/JB.185.9.2879-2886.2003 PMC154395

[51] Franke GC, Dobinsky S, Mack D, Wang CJ, Sobottka I, Christner M, et al Expression and functional characterization of gfpmut3.1 and its unstable variants in *Staphylococcus epidermidis* . J Microbiol Methods 2007 Nov;71(2):123–32. 17919756 10.1016/j.mimet.2007.08.015

[52] Knobloch JK, Jager S, Horstkotte MA, Rohde H, Mack D. RsbU-dependent regulation of *Staphylococcus epidermidis* biofilm formation is mediated via the alternative sigma factor sigmaB by repression of the negative regulator gene *icaR* . Infect Immun 2004 Jul;72(7):3838–48. 15213125 10.1128/IAI.72.7.3838-3848.2004 PMC427440

[53] Rieu I, Powers SJ. Real-time quantitative RT-PCR: design, calculations, and statistics. Plant Cell 2009 Apr;21(4):1031–3. 10.1105/tpc.109.066001 19395682 PMC2685626

[54] Knobloch JK, Jager S, Huck J, Horstkotte MA, Mack D. *mecA* is not involved in the sigmaB-dependent switch of the expression phenotype of methicillin resistance in *Staphylococcus epidermidis* . Antimicrob Agents Chemother 2005 Mar;49(3):1216–9. 15728932 10.1128/AAC.49.3.1216-1219.2005 PMC549230

[55] Pfaffl MW. A new mathematical model for relative quantification in real-time RT-PCR. Nucleic Acids Res 2001 May 1;29(9):e45 11328886 10.1093/nar/29.9.e45 PMC55695

[56] Pozzi C, Waters EM, Rudkin JK, Schaeffer CR, Lohan AJ, Tong P, et al Methicillin resistance alters the biofilm phenotype and attenuates virulence in *Staphylococcus aureus* device-associated infections. PLoS Pathog 2012;8(4):e1002626 10.1371/journal.ppat.1002626 22496652 PMC3320603

[57] Malone CL, Boles BR, Lauderdale KJ, Thoendel M, Kavanaugh JS, Horswill AR. Fluorescent reporters for *Staphylococcus aureus*. J Microbiol Methods 2009 Mar 3. 10.1016/j.mimet.2009.02.011 PMC2693297 19264102

[58] Handke LD, Slater SR, Conlon KM, O'Donnell ST, Olson ME, Bryant KA, et al SigmaB and SarA independently regulate polysaccharide intercellular adhesin production in *Staphylococcus epidermidis* . Can J Microbiol 2007 Jan;53(1):82–91. 17496953 10.1139/w06-108

[59] Olson ME, Todd DA, Schaeffer CR, Paharik AE, Van Dyke MJ, Buttner H, et al. The Staphylococcus epidermidis agr quorum-sensing system: signal identification, cross-talk, and importance in colonization. J Bacteriol 2014 Jul 28;JB-14. 10.1128/JB.01882-14 PMC4187671 25070736

