# Supplementary figures and images for: An 18 kDa Scaffold Protein Is Critical for Staphylococcus epidermidis Biofilm Formation
Source: PLoS Pathog. 2015 Mar 23;11(3):e1004735. doi: 10.1371/journal.ppat.1004735 (PMC4370877; doi:10.1371/journal.ppat.1004735)

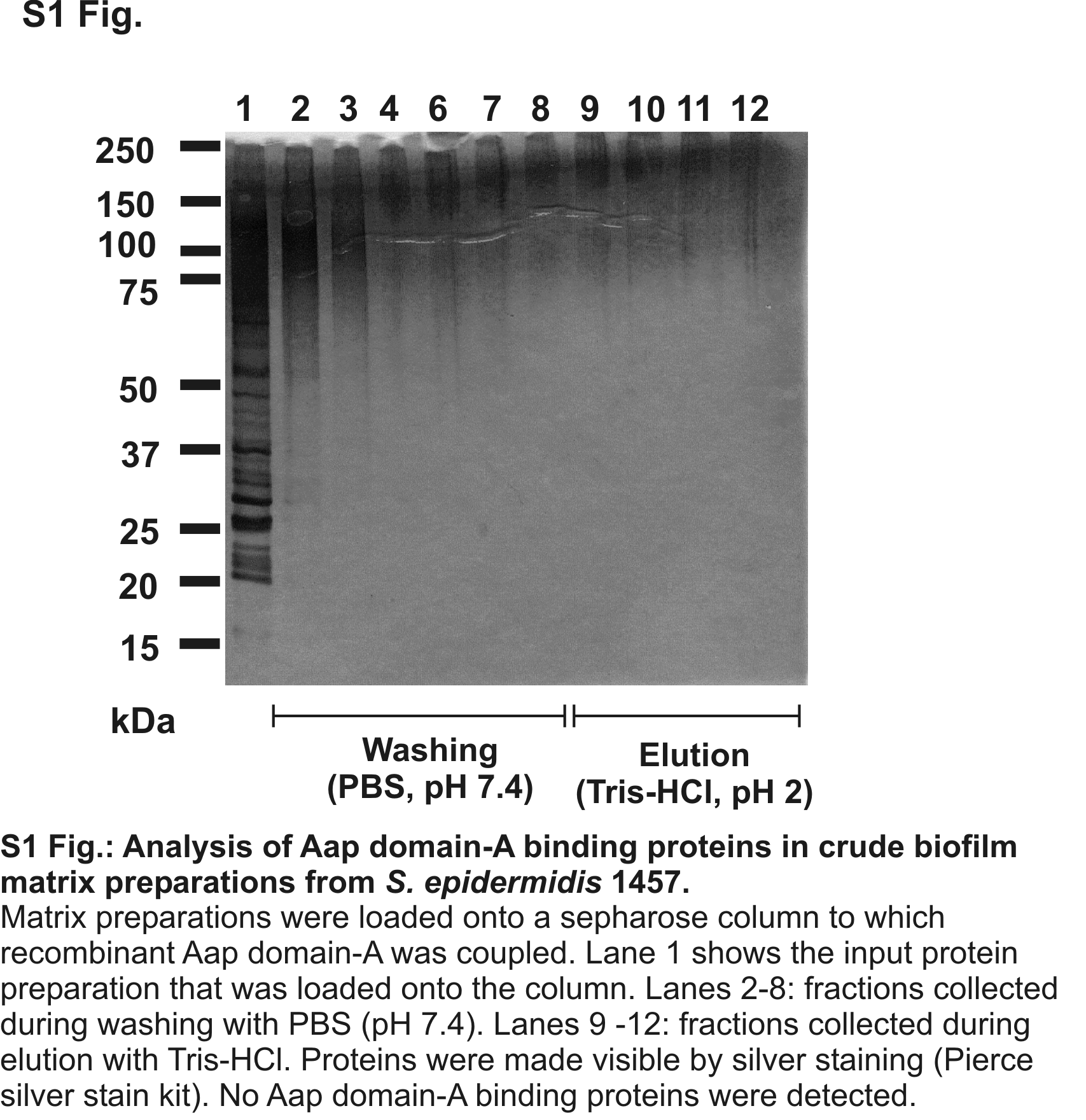

Supplement: S1 Fig — Matrix preparations were loaded onto a sepharose column to which recombinant Aap domain-A was coupled. Lane 1 shows the input protein preparation that was loaded onto the column. Lanes 2–8: fractions collected during washing with PBS (pH 7.4). Lanes 9–12: fractions collected during elution with Tris-HCl. Proteins were made visible by silver staining (Pierce silver stain kit). No Aap domain-A binding proteins were detected. (TIF) [file ppat.1004735.s001.tif]

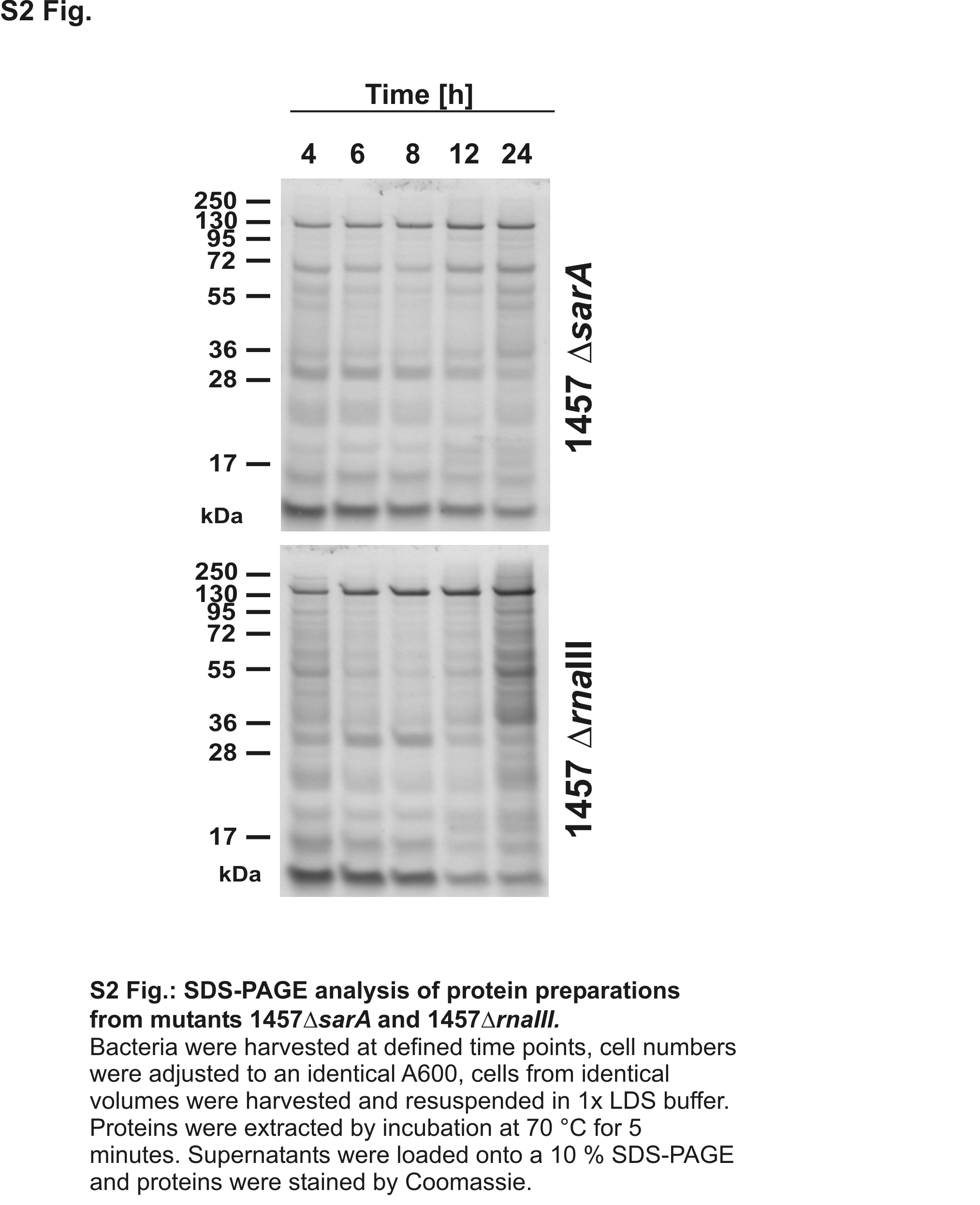

Supplement: S2 Fig — Bacteria were harvested at defined time points, cell numbers were adjusted to an identical A600, and cells from identical volumes were harvested and resuspended in 1x LDS buffer. Proteins were extracted by incubation at 70°C for 5 minutes. Supernatants were loaded onto a 10% SDS-PAGE and proteins were stained by Coomassie. (TIF) [file ppat.1004735.s002.tif]

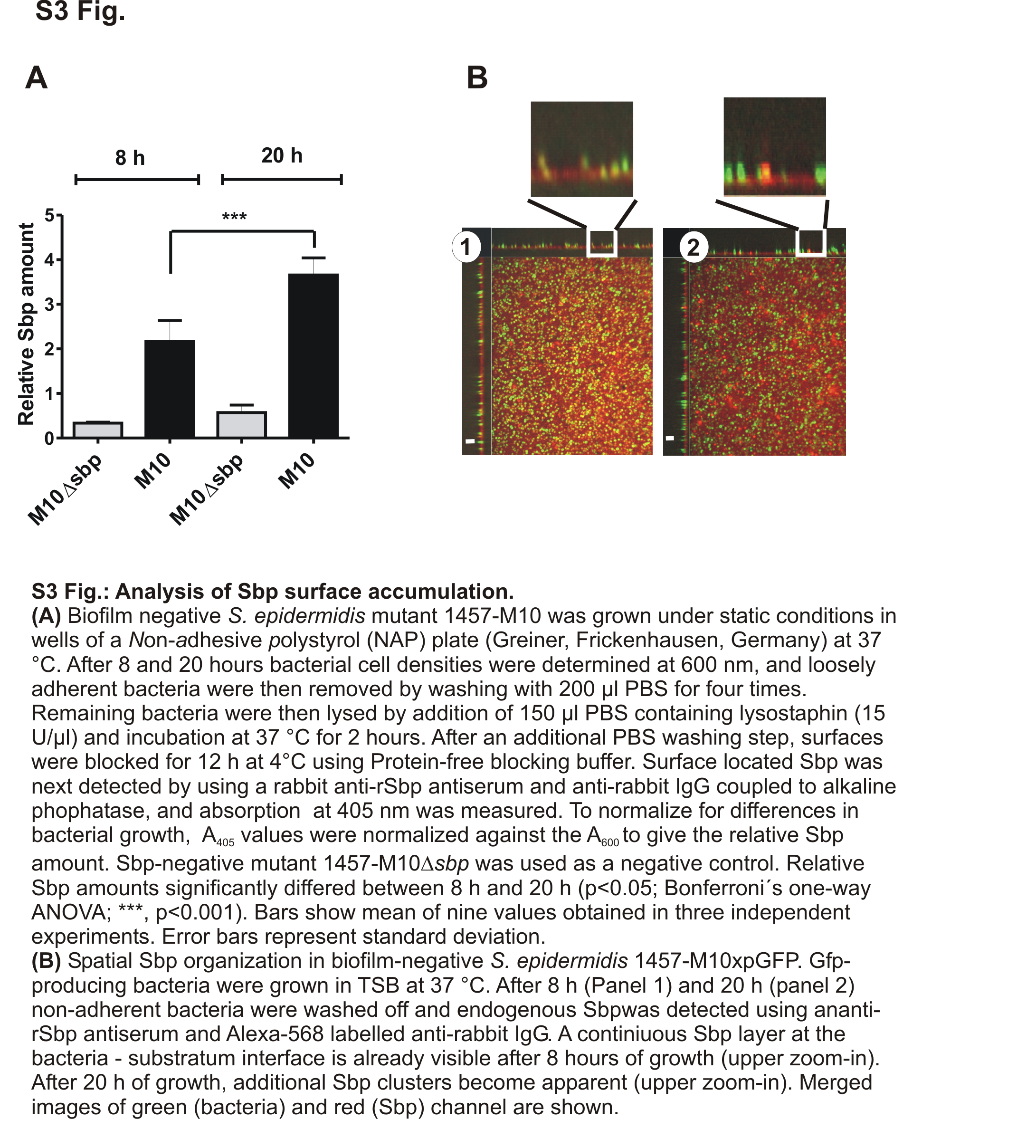

Supplement: S3 Fig — (A). Biofilm negative S. epidermidis mutant 1457-M10 was grown under static conditions in wells of a Non-adhesive polystyrol (NAP) plate (Greiner, Frickenhausen, Germany) at 37°C. After 8 and 20 hours bacterial cell densities were determined at 600 nm, and loosely adherent bacteria were then removed by washing with 200 μl PBS for four times. Remaining bacteria were then lysed by addition of 150 μl PBS containing lysostaphin (15 U/μl) and incubation at 37°C for 2 hours. After an additional PBS washing step, surfaces were blocked for 12 h at 4°C using Protein-free blocking buffer. Surface located Sbp was next detected by using a rabbit anti-rSbp antiserum and anti-rabbit IgG coupled to alkaline phosphatase, and absorption at 405 nm was measured. To normalize for differences in bacterial growth, A405 values were normalized against the A600 to give the relative Sbp amount. Sbp-negative mutant 1457-M10Δsbp was used as a negative control. Relative Sbp amounts significantly differed between 8 h and 20 h (p<0.05; Bonferroni’s one-way ANOVA; ***, p<0.001). Bars show mean of nine values obtained in three independent experiments. Error bars represent standard deviation. (B) Spatial Sbp organization in biofilm-negative S. epidermidis 1457-M10(pGFP). Gfp-producing bacteria were grown in TSB at 37°C. After 8 h (Panel 1) and 20 h (panel 2) non-adherent bacteria were washed off and endogenous Sbp was detected using an anti-rSbp antiserum and Alexa-568 labelled anti-rabbit IgG. A continuous Sbp layer at the bacteria—substratum interface is already visible after 8 hours of growth (upper zoom-in). After 20 h of growth, additional Sbp clusters become apparent (upper zoom-in). Merged images of green (bacteria) and red (Sbp) channel are shown. (TIF) [file ppat.1004735.s003.tif]

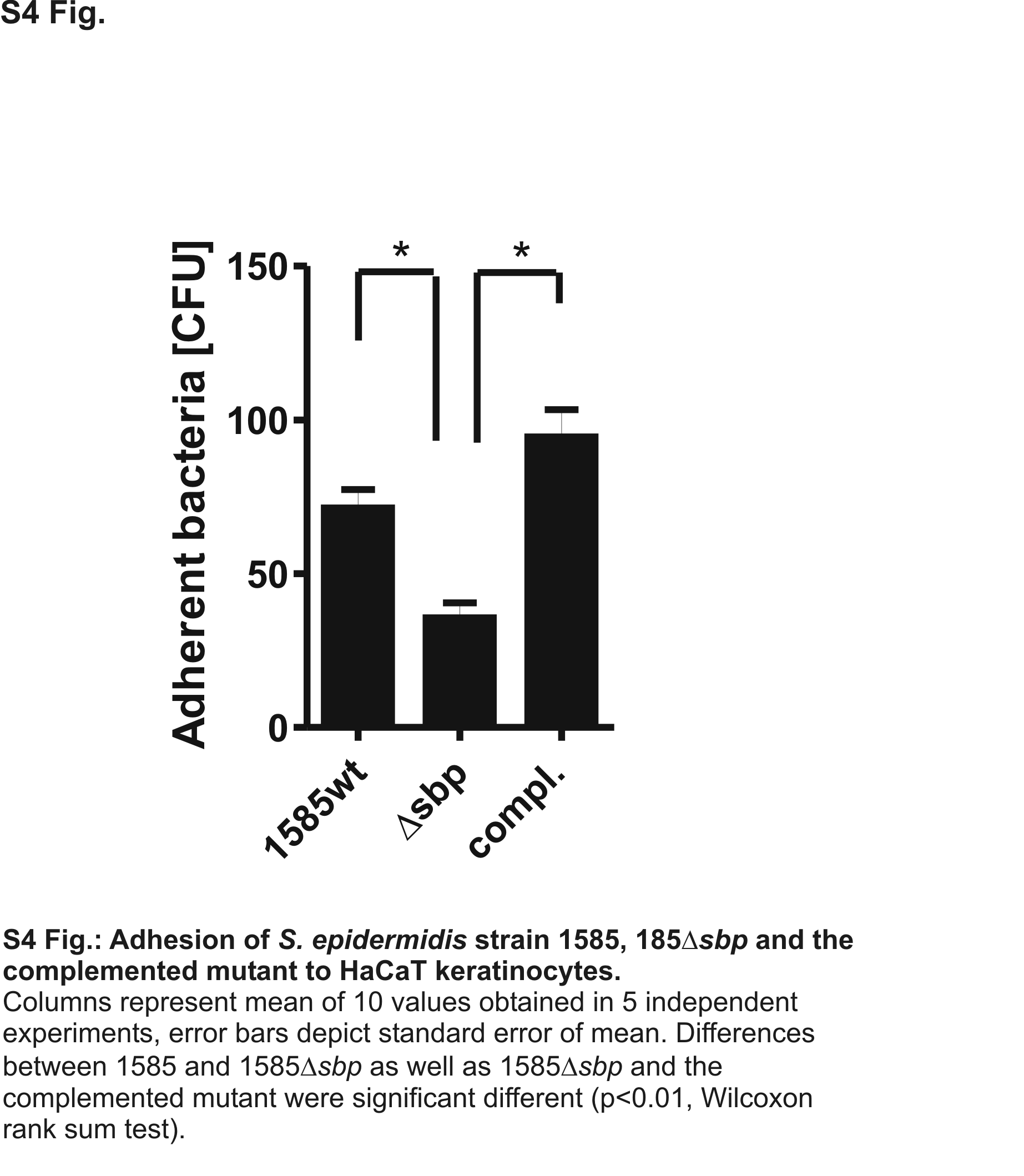

Supplement: S4 Fig — Columns represent mean of 10 values obtained in 5 independent experiments, error bars depict standard error of mean. Differences between 1585 and 1585Δsbp as well as 1585Δsbp and the complemented mutant were significant different (p<0.01, Wilcoxon rank sum test). (TIF) [file ppat.1004735.s004.tif]

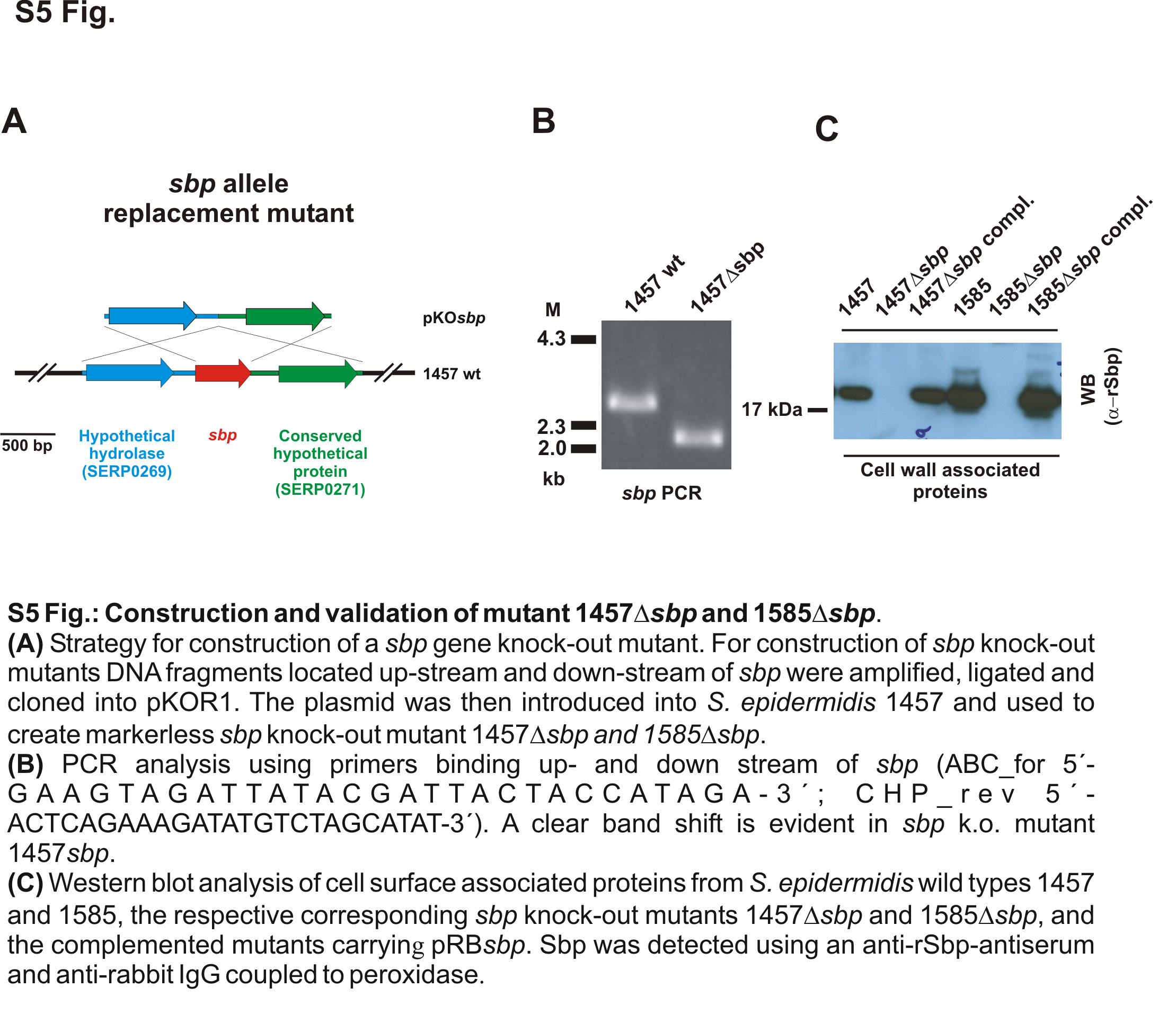

Supplement: S5 Fig — (A) Strategy for construction of a sbp gene knock-out mutant. For construction of sbp knock-out mutants DNA fragments located up-stream and down-stream of sbp were amplified, ligated and cloned into pKOR1. The plasmid was then introduced into S. epidermidis 1457 and used to create markerless sbp knock-out mutant 1457Δsbp. (B) PCR analysis using primers binding up- and downstream of sbp (ABC_for 5′-GAAGTAGATTATACGATTACTACCATAGA-3′; CHP_rev 5′-ACTCAGAAAGATATGTCTAGCATAT-3′). A clear band shift is evident in sbp k.o. mutant 1457Δsbp. (C) Western blot analysis of cell surface associated proteins from S. epidermidis wild types 1457 and 1585, the respective corresponding sbp knock-out mutants 1457Δsbp and 1585Δsbp, and the complemented mutants carrying pRBsbp. Sbp was detected using an anti-rSbp-antiserum and anti-rabbit IgG coupled to peroxidase. (TIF) [file ppat.1004735.s005.tif]

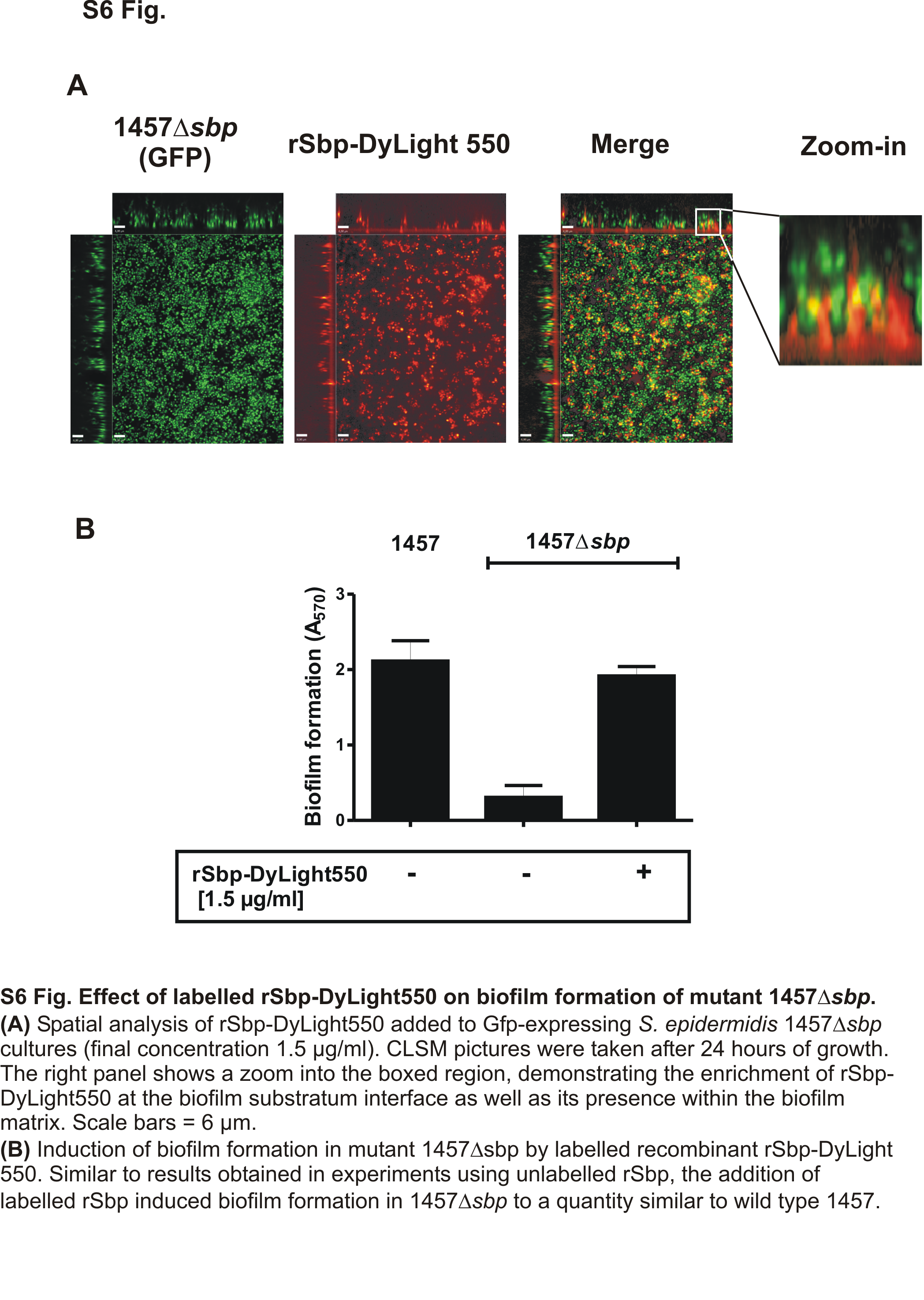

Supplement: S6 Fig — (A) Spatial analysis of rSbp-DyLight550 added to Gfp-expressing S. epidermidis 1457Δsbp cultures (final concentration 1.5 μg/ml). CLSM pictures were taken after 24 hours of growth. The right panel shows a zoom into the boxed region, demonstrating the enrichment of rSbp-DyLight550 at the biofilm substratum interface as well as its presence within the biofilm matrix. Scale bars = 6 μm. (B) Induction of biofilm formation in 1457Δsbp by labelled recombinant rSbp-DyLight 550. Similar to results obtained in experiments using unlabelled rSbp, the addition of labelled rSbp induced biofilm formation in 1457Δsbp to a quantity similar to wild type 1457. (TIF) [file ppat.1004735.s006.tif]

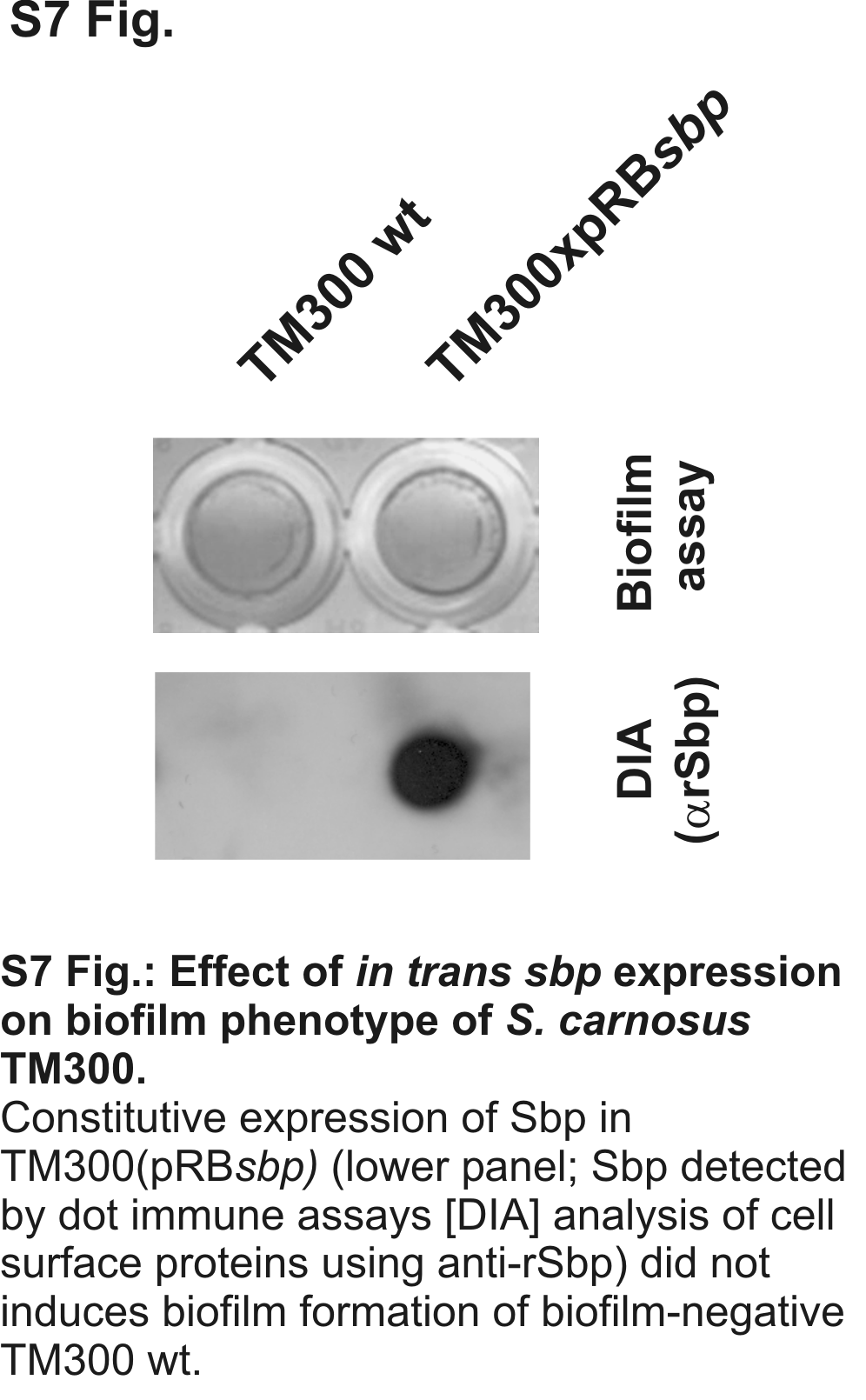

Supplement: S7 Fig — Constitutive expression of Sbp in TM300(pRBsbp) (lower panel; Sbp detected by dot immune assays [DIA] analysis of cell surface proteins using anti-rSbp) did not induce biofilm formation of biofilm-negative TM300 wt. (TIF) [file ppat.1004735.s007.tif]

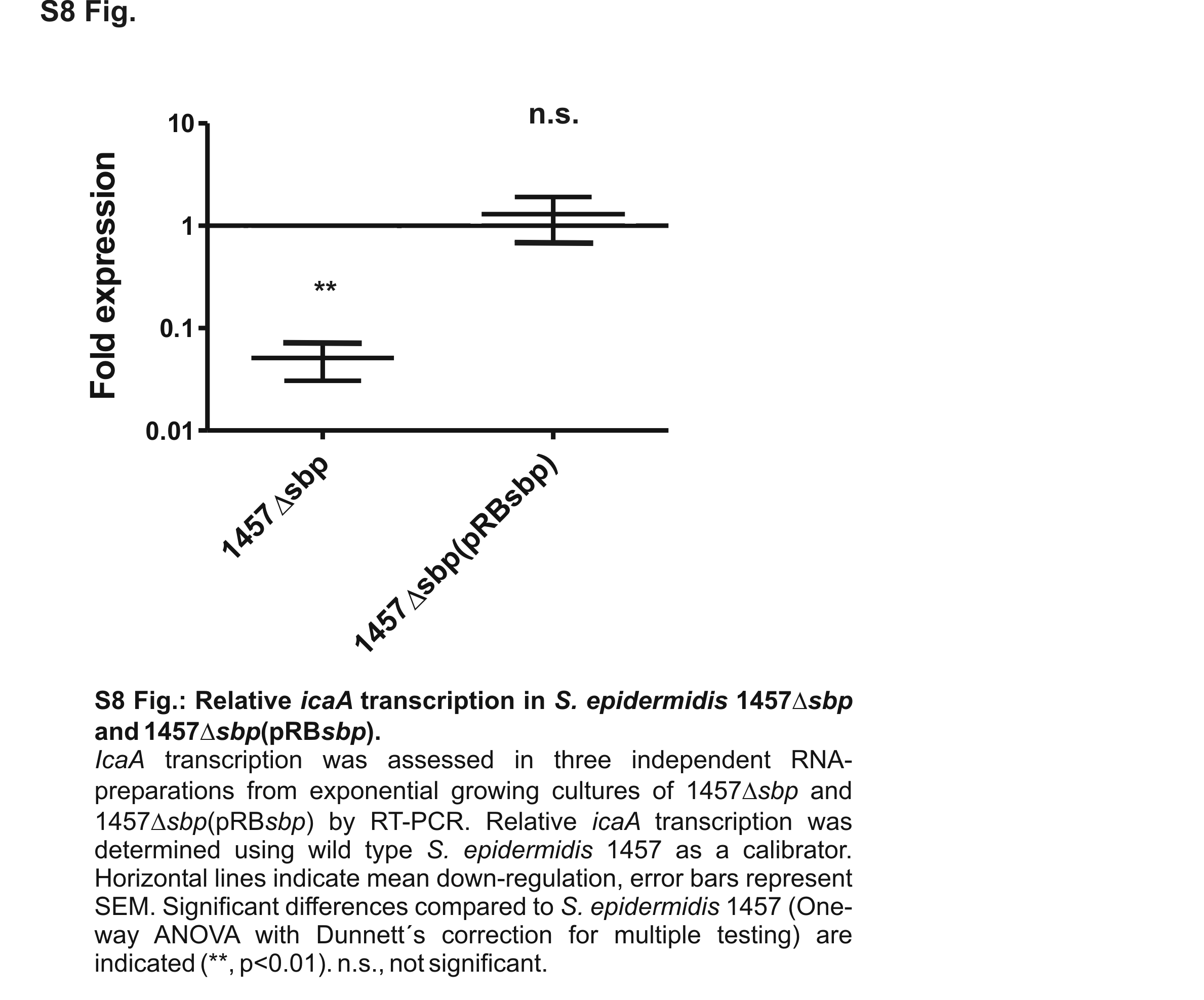

Supplement: S8 Fig — icaA transcription was assessed in three independent RNA-preparations from exponential growing cultures of 1457Δsbp and 1457Δsbp(pRBsbp) by RT-PCR. Relative icaA transcription was determined using wild type S. epidermidis 1457 as a calibrator. Horizontal lines indicate mean down-regulation, error bars represent SEM. Significant differences compared to S. epidermidis 1457 (One-way ANOVA with Dunnett’s correction for multiple testing) are indicated (**, p<0.01). n.s., not significant. (TIF) [file ppat.1004735.s008.tif]

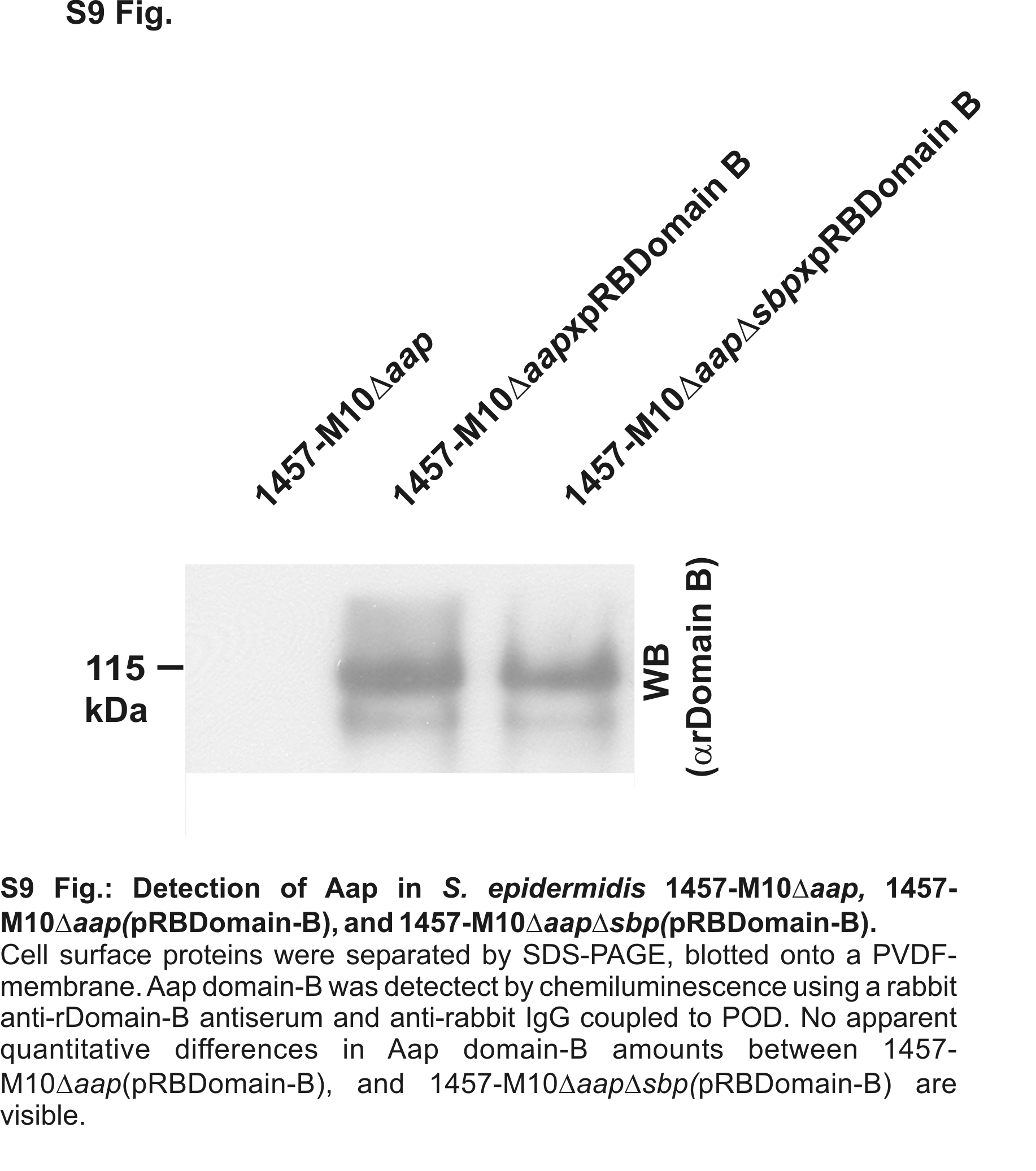

Supplement: S9 Fig — Cell surface proteins were separated by SDS-PAGE, blotted onto a PVDF-membrane. Aap domain-B was detectect by chemiluminescence using a rabbit anti-rDomain-B antiserum and anti-rabbit IgG coupled to POD. No apparent quantitative differences in Aap domain-B amounts between 1457-M10Δaap(pRBDomain-B), and 1457-M10ΔaapΔsbp(pRBDomain-B) are visible. (TIF) [file ppat.1004735.s009.tif]

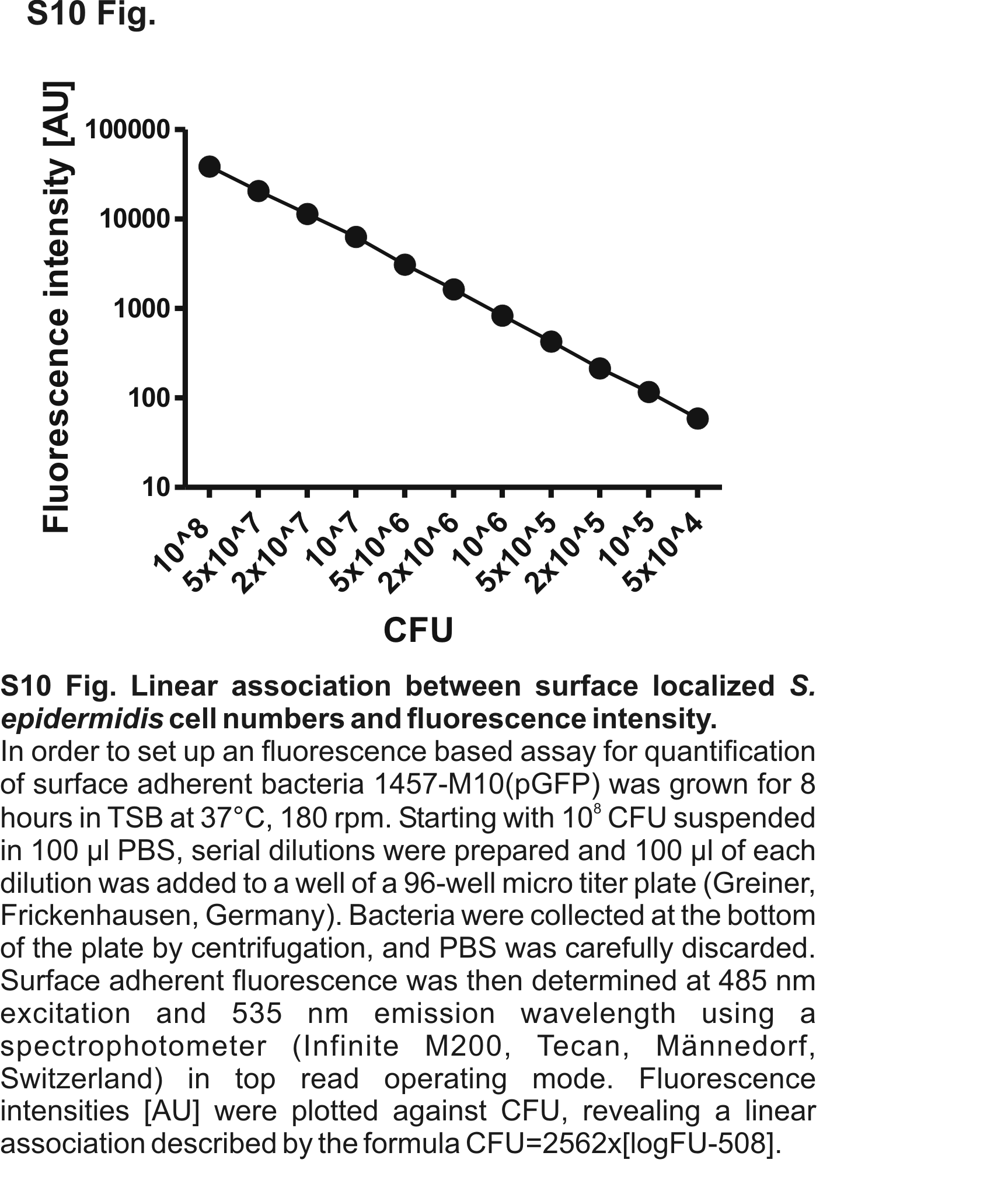

Supplement: S10 Fig — In order to set up an fluorescence based assay for quantification of surface adherent bacteria 1457-M10(pGFP) was grown for 8 hours in TSB at 37°C, 180 rpm. Starting with 108 CFU suspended in 100 μl PBS, serial dilutions were prepared and 100 μl of each dilution was added to a well of a 96 well micro titer plate (Greiner, Frickenhausen, Germany). Bacteria were collected at the bottom of the plate by centrifugation, and PBS was carefully discarded. Surface adherent fluorescence was then determined at 485 nm excitation and 535 nm emission wavelength using a spectrophotometer (Infinite M200, Tecan, Männedorf, Switzerland) in top read operating mode. Fluorescence intensities [AU] were plotted against CFU, revealing a linear association described by the formula CFU = 2562x[logFU-508]. (TIF) [file ppat.1004735.s010.tif]
